# *De Novo* Design of Functional Coassembling Organic–Inorganic Hydrogels for Hierarchical Mineralization and Neovascularization

**DOI:** 10.1021/acsnano.0c09814

**Published:** 2021-06-28

**Authors:** Babatunde O. Okesola, Ana Karen Mendoza-Martinez, Gianluca Cidonio, Burak Derkus, Delali K. Boccorh, David Osuna de la Peña, Sherif Elsharkawy, Yuanhao Wu, Jonathan I. Dawson, Alastair W. Wark, Dafna Knani, Dave J. Adams, Richard O. C. Oreffo, Alvaro Mata

**Affiliations:** † Institute of Bioengineering, Queen Mary University of London, London E1 4NS, U.K.; ‡ School of Engineering and Materials Science, Queen Mary University of London, London E1 4NS, U.K.; § Bone and Joint Research Group, Centre for Human Development, Stem Cells and Regeneration, Institute of Developmental Sciences, University of Southampton, Southampton SO16 6YD, U.K.; ∥ Department of Chemistry, Faculty of Science, Ankara University, 06560 Ankara, Turkey; ⊥ Department of Pure and Applied Chemistry, Technology and Innovation Centre, University of Strathclyde, Glasgow G1 1RD, U.K.; # Centre for Oral, Clinical, and Translational Sciences, Faculty of Dentistry, Oral, and Craniofacial Sciences, King’s College London, London SE1 1UL, U.K.; ∇ School of Pharmacy, University of Nottingham, Nottingham NG7 2RD, U.K.; □ Biodiscovery Institute, University of Nottingham, Nottingham NG7 2RD, U.K.; ○ Department of Biotechnology Engineering, ORT Braude College, Karmiel 2161002, Israel; △ School of Chemistry, College of Science and Engineering, University of Glasgow, Glasgow G12 8QQ, U.K.; @ Department of Chemical and Environmental Engineering, University of Nottingham, Nottingham NG7 2RD, U.K.; ◆ Center for Life Nano- & Neuro- Science (CL2NS), Fondazione Istituto Italiano di Tecnologia, 00161 Rome, Italy

**Keywords:** laponite, nanocomposite hydrogels, coassembly, supramolecular, biomineralization, peptide amphiphiles, multicomponent biomaterials

## Abstract

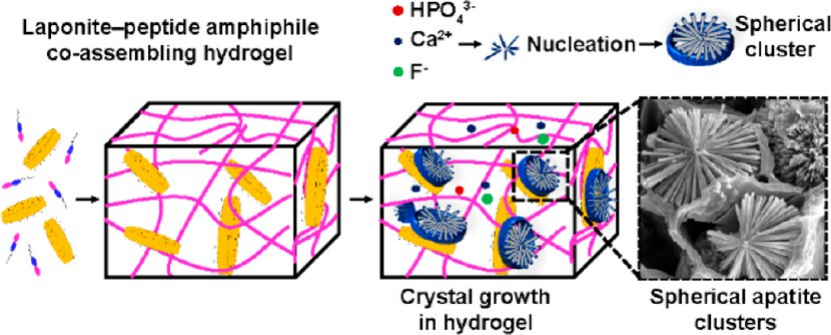

Synthetic nanostructured materials incorporating both organic and inorganic components offer a unique, powerful, and versatile class of materials for widespread applications due to the distinct, yet complementary, nature of the intrinsic properties of the different constituents. We report a supramolecular system based on synthetic nanoclay (Laponite, **Lap**) and peptide amphiphiles (PAs, **PAH3**) rationally designed to coassemble into nanostructured hydrogels with high structural integrity and a spectrum of bioactivities. Spectroscopic and scattering techniques and molecular dynamic simulation approaches were harnessed to confirm that **PAH3** nanofibers electrostatically adsorbed and conformed to the surface of **Lap** nanodisks. Electron and atomic force microscopies also confirmed an increase in diameter and surface area of **PAH3** nanofibers after coassembly with **Lap**. Dynamic oscillatory rheology revealed that the coassembled **PAH3-Lap** hydrogels displayed high stiffness and robust self-healing behavior while gas adsorption analysis confirmed a hierarchical and heterogeneous porosity. Furthermore, this distinctive structure within the three-dimensional (3D) matrix provided spatial confinement for the nucleation and hierarchical organization of high-aspect ratio hydroxyapatite nanorods into well-defined spherical clusters within the 3D matrix. Applicability of the organic–inorganic **PAH3-Lap** hydrogels was assessed *in vitro* using human bone marrow-derived stromal cells (hBMSCs) and *ex vivo* using a chick chorioallantoic membrane (CAM) assay. The results demonstrated that the organic–inorganic **PAH3-Lap** hydrogels promote human skeletal cell proliferation and, upon mineralization, integrate with the CAM, are infiltrated by blood vessels, stimulate extracellular matrix production, and facilitate extensive mineral deposition relative to the controls.

## Introduction

Nature contains an array of functional nanomaterials that result from the supramolecular coassembly of organic and inorganic building blocks across multiple length scales. Materials such as tooth enamel, bones, nacre from mollusc shells, and marine diatom frustules exhibit a high level of precision over their molecular composition, hierarchical structure, and morphology. The inherent characteristics endow these nanomaterials with properties ranging from high stiffness to light-emission.^1,2^ A fundamental characteristic of natural organic–inorganic composites is the presence of organic matrixes exhibiting ordered arrays of confined charged groups, which induce and regulate the spatial nucleation and hierarchical organization of crystals.^3,4^ These organic components are generally 3D hydrogel-like materials made from multiple components such as proteins, peptides, polyamines, and polysaccharides.^5,6^

This bottom-up “nanofabrication” strategy employed by nature has been harnessed in materials science to design organic–inorganic multicomponent hydrogels with innovative properties.^2,7^ In particular, significant research efforts have been expended to integrate the intrinsic electrical conductivity, magnetism, adhesiveness, and hardness of inorganic nanomaterials^8^ with the inherent functionality of both natural (*e.g.*, collagen,^9^ elastin,^10^ DNA,^11^ and hyaluronic acid^12^) and synthetic (*e.g.*, dibenzylidene-d-sorbitol,^8,13^ peptides,^7^ and polymers^14^) molecules in the design of advanced organic–inorganic hydrogels. These organic–inorganic multicomponent hydrogels are attractive platforms for a wide range of applications in optics, microelectronics, energy storage, catalysis, sensing/environmental cleanup, and nanomedicine.^15^ However, the resulting structures and functions exhibited by these composite materials remain far from those of the natural organic–inorganic materials.^16^

To enhance the properties of organic–inorganic nanocomposites, co-organization of two or more types of inorganic components within the same nanoscale object provides an opportunity to prepare higher-ordered nano-objects with synergistic properties.^17^ Thus, application of such an inorganic approach takes advantage of the distinct properties of the individual inorganics as well as the emergence of new ones that result from their interactions. Current strategies for fabricating organic–inorganic nanocomposites with multi-inorganic nano-objects are driven by either programmed assembly or reaction-diffusion mechanisms. Programmed assembly involves molecular recognition-driven interparticle aggregation. For example, in a seminal work by Mann and co-workers, DNA-directed attachment of gold nanoparticles to single nanoparticles of silica was used to fabricate discrete nano-objects.^18^ Similarly, barstar-capped iron oxide nanoparticles and barnase-coated quantum dot nanoparticles were coassembled to create superstructures with magnetofluorescence properties.^19^ Other approaches using complementary streptavidin/biotin or antibody/antigen have been harnessed to integrate multiple inorganics in a single organic–inorganic nanocomposite.^20^ In contrast, systems based on reaction-diffusion mechanisms enable assembly of inorganics into nano-objects with spatiotemporal orientation, not readily accessible by equilibrium processes.^17,19^ Examples have been demonstrated in biomineralization,^21^ microfabrication,^22−27^ formation of microlenses,^28^ and dynamic materials.^29^ Reactions of inorganic species, coupled with diffusion in hydrogel media, can lead to the formation of nano-objects with structural hierarchy and complexity as well as multifunctional properties. The rate of formation of these nano-objects within a hydrogel can be controlled by fluid flow, spontaneous compartmentalization, diffusive transport, and Ostwald ripening.^30^

Self-assembling peptides are particularly attractive platforms for the design of organic–inorganic nanostructures, given their intrinsic propensity to assemble into 3D hydrogels, comprising well-defined nanostructures and an ability to display tunable binding affinity for inorganic nanostructures.^31^ These unique attributes of self-assembling peptides have been harnessed to fabricate diverse peptide–inorganic hybrid materials with impressive properties and functionalities. The ability to harness the spatiotemporal organization and enhanced surface chemistry of peptide–inorganic hydrogels would represent a step-change platform to guide crystal morphogenesis in 3D confinement.^7^

Laponite XLG (Lap), a trioctahedral synthetic hectorite (Na^+^_0.7_[(Si_8_Mg_5.5_Li_0.3_)O_20_(OH)_4_]^−0.7^), is a particularly important class of nanosilicate being explored for the design of functional nanomaterials.^32^ Lap displays an ultrathin 2D nanostructure (diameter = 25–30 nm and thickness <1 nm), discotic charged surface (permanent negative charge on the surface and positive rim charge), high specific surface area (800 m^2^/g), and optical transparency.^33^ Consequently, Lap has been coassembled with synthetic polymers,^34−36^ DNA,^37^ or proteins^9,38^ to develop organic–inorganic hydrogels for numerous biomedical applications and additive manufacturing.^32^ For example, there is a growing interest in the use of polymer–Laponite nanocomposite hydrogels as injectable vehicles for biological cargo including cells,^39^ drug molecules,^40^ and growth factors,^41^ because of their intrinsic shear-thinning property. However, synthetic polymers typically require complex chemical synthesis and purification steps and lack a well-defined structure–property relationship, while natural polymers lack structural tunability and can be difficult to obtain. Therefore, the use of modular and easy-to-synthesize organic building blocks, such as self-assembling peptides, can serve as simpler and more predictable components to interact with and guide the assembly of Lap. Peptide amphiphiles (PAs), a class of self-assembling peptides, have been engineered to facilitate coassembly with biomolecules such as hyaluronic acid,^42^ elastin-like polypeptides,^43^ keratin,^44^ resilin-like polypeptide,^45^ as well as nonpeptidic molecules^46^ to generate different architectures with structural hierarchy and enhanced mechanical and functional properties.

**Figure 1:**
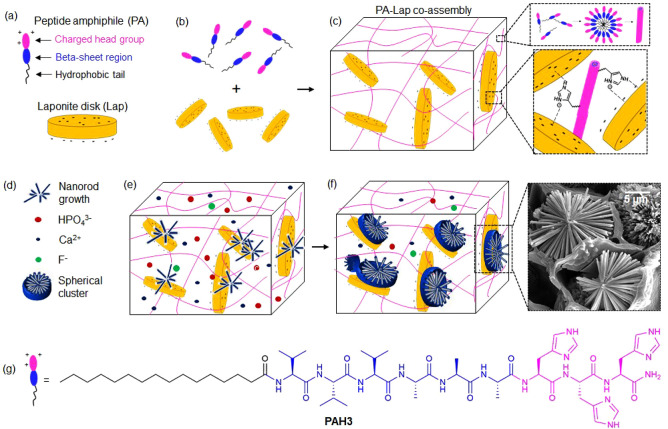
Supramolecular coassembly of exfoliated Lap nanodisks (−ve) and PA (+ve) to create 3D hydrogels able to guide nucleation and hierarchical growth of hydroxyapatite crystals. (a) Structural representation of a PA with its domains and a Lap nanodisk. (b) Supramolecular coassembly of PAs and Lap to create (c) mechanically robust organic–inorganic hybrid hydrogels with interconnected nanofibers physically cross-linked by Lap nanodisks. Diffusion of (d) mineralizing ionic species into the 3D organic–inorganic hybrid hydrogels triggers the (e) nucleation and (f) hierarchical crystal growth of hydroxyapaptite crystals into high-aspect ratio nanorods organized in spherical clusters. (g) Structural formula for histidine-based PAs.

Herein, we report an organic–inorganic nanocomposite hydrogel based on the coassembly of Lap nanodisks with PAs. The hydrogels displayed high mechanical strength, shear-thinning behavior, and molecular diversity. Furthermore, the resulting PA-Lap coassembled structures served as spatial confinements to guide the formation of nanocrystals with well-defined morphologies across multiple length scales, leading to the formation of multi-inorganic–organic nano-objects (schematically illustrated in Figure 1). These mineralized hydrogels supported cell adhesion, proliferation, differentiation, and neovascularization as assessed by *in vitro* cell culture and *ex vivo* using a chick chorioallantoic membrane (CAM) assay.

## Results and Discussion

### Rationale of the Material Design

Our system aims to harness the intrinsic discotic and surface anisotropy of Lap nanodisks and the modularity and self-assembling capacity of PAs to engineer robust and biocompatible hydrogels that not only exhibit the properties of each component but, critically, emergent properties as a result of their coassembly. The PA (**PAH3**) is a histidine-rich molecule (CH_3_-(CH_2_)_14_-CONH-VVVAAAHHH-CONH_2_, Figure 1g) and is designed to coassemble through interaction with **Lap**. The unique aromatic imidazole side chain of histidine is key to the self-assembly of proteinaceous fibers driven by organic–inorganic complexation, which is known to generate self-healable and mechanically reinforced biogenic architectures.^47^ We reasoned that the histidine aromatic imidazole side chain (p*K*_a_ ∼ 6.0), which becomes cationic in mildly acidic conditions, would promote electrostatic and intercalation interactions between **PAH3** and the negatively charged **Lap** disk surfaces (surface adsorption). Based on the pioneering work of Aida and co-workers on coassembling Lap nanodisks with guanidinium-based dendritic binders,^48^ we hypothesized that our **PAH3-Lap** coassembling system would generate mechanically reinforced organic–inorganic hydrogels. It is noteworthy that although cationic PAs with lysine charged head groups have been extensively exploited, self-assembly and gelation of histidine-based **PAH3** have yet to be explored. **PAK3** (CH_3_-(CH_2_)_14_-CONH-VVVAAAKKK-CONH_2_) and **PAE3** (CH_3_-(CH_2_)_14_-CONH-VVVAAAEEE-CONH_2_) were used as controls throughout the experiments.

### Mechanism of PAH3-Lap Supramolecular Coassembly

#### Electrostatic and Sergeant–Soldier Interactions Drive Coassembly

PAs were designed and synthesized as previously reported.^49^ To assess electrostatic interactions between **PAH3** and **Lap** nanodisks, we measured the zeta potential (ζ) values and hydrodynamic radii (Rh) of individual components against their mixture. The interaction between **Lap** (ζ = −35 mV, Rh = 36.42 ± 2.01 nm) and **PAH3** (ζ = +25 mV, Rh = 80.11 ± 4.31 nm) evidently revealed the formation of a higher-ordered nanostructure with an increased hydrodynamic radius (ζ = −10 mV, Rh = 115.11 ± 6.54 nm) (Supporting Information Figure S1a–e). In contrast, the control **PAE3** (−30 mV), which exhibits similar net charge as **Lap**, did not display any interaction, suggesting that the formation of **PAH3-Lap** was at least partly driven by electrostatic interactions.

**Figure 2:**
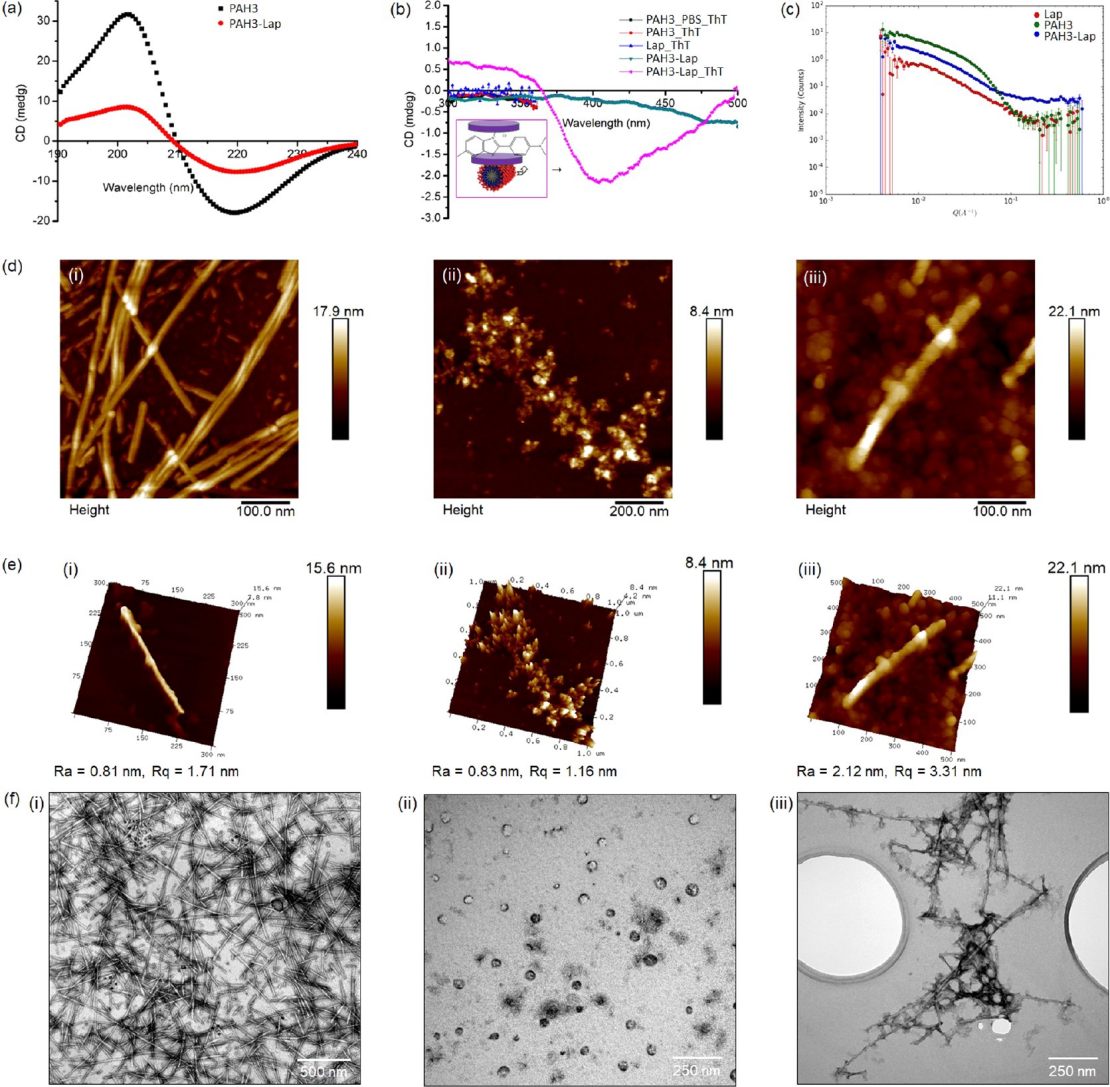
Structural characterization of supramolecular coassembly. (a) CD spectra of an aqueous solution of **PAH3** before (square traces) and after (circular traces) adding **Lap**. (b) Induced CD spectra of thioflavin T (ThT) in the presence of **PAH3** in PBS 1x, **PAH3-Lap** partial hydrogels, and aqueous solution of **Lap**. The inset represents the proposed mechanism for chirality transfer from **PAH3** to **Lap** nanodisk as a result of supramolecular coassembly, which was confirmed with the use of the molecular rotor ThT. (c) Synchrotron small-angle neutron scattering of **PAH3**, **Lap**, and **PAH3-Lap** coassembly. (d) Atomic force micrographs of (i) **PAH3**, (ii) **Lap**, and (iii) **PAH3-Lap** coassembly as well as the (e) surface topography profile for (i) **PAH3**, (ii) **Lap**, and (iii) **PAH3-Lap** coassembly. (f) Transmission electron micrographs of aqueous suspension of (i) **PAH3,** (ii) **Lap**, and (iii) **PAH3-Lap**.

Given that PAs are generally known to self-assemble into β-sheets, we used circular dichroism (CD) spectroscopy to assess interaction between **PAH3** and **Lap** nanodisks. The CD measurements revealed that **PAH3** displays a typical β-sheet conformation with an absorption maximum and minimum at 202 and 218 nm, respectively (Figure 2a). Upon coassembly with **Lap**, the CD intensities at 202 and 218 nm decreased by ∼16 and ∼11 mdeg, respectively. This significant decrease in CD intensities might be due to strong surface adhesion of **PAH3** to the **Lap** nanodisks, resulting in a significant disruption of the **PAH3** β-sheet conformation. Such disruption has been previously reported in silk molecules upon interaction with Lap.^37^ To further confirm surface adsorption of **PAH3** to **Lap**, we used a standard molecular probe thioflavin T (ThT) which is known to monitor PA self-assembly in aqueous environments.^46^ When the achiral ThT was introduced into a diluted **PAH3-Lap** hydrogel, a negative band at 410 nm, which is indicative of bound ThT, became apparent (Figure 2b). This absorption band was not observed when ThT was mixed with **PAH3** in water, **PAH3** in PBS, or **Lap** in water. Although ThT coassembled with the **Lap** nanodisk suspension, the Lap–ThT complex exhibited no CD signal, suggesting that ThT acquired an induced chirality due to surface adsorption to Lap nanodisks templated by **PAH3** nanofibers (Figure 2b inset). Such nanofiber templating of **Lap** nanodisks has been previously demonstrated with collagen nanofibers due to electrostatic interactions between the positively charged amino acid groups on the periphery of the collagen nanofibrils and negatively charged **Lap** surfaces.^50^

#### Nanoscopic Evidence of PA-Lap Coassembly

Nanoscale characterization of the **PAH3-Lap** composites also confirmed supramolecular integration of both components to generate a higher-ordered nanostructure. First, we used synchrotron small-angle neutron scattering (SANS) to characterize the individual components (**Lap** and **PAH3**), as well as their mixture (**PAH3-Lap**). The SANS data for **Lap** alone in deuterium oxide (D_2_O) possesses a Q^–1.8^ dependency in the range 0.01 < 0 < 0.1 Å^–1^, which is consistent with a thin disk-shaped structure with a thickness and diameter of ∼12 and ∼256 Å, respectively (Figure 2c, Supporting Information Table S1). The SANS data for **PAH3** alone in D_2_O shows the existence of cylinder-like nanostructures with a radius of ∼38 Å and several microns in length. The scattering profile of the mixture of **Lap** and **PAH3** shows the coexistence of both disk-like nanostructures and cylindrical nanofibers, suggesting a supramolecular coassembly of both nanostructures. The radius of **PAH3** nanofibers increased by ∼45 Å after coassembly with **Lap**, suggesting that both components coassembled to form a higher-ordered nanostructure consisting of cylindrical nanofibers and nanodisks. We also confirmed coexistence of **Lap** disks and **PAH3** nanofibers by atomic force microscopy (AFM) (Figure 2d_i–iii). The corresponding surface roughness parameters Ra and Rq for the **PAH3-Lap** nanocomposites (Ra = 2.12 nm, Rq = 3.31 nm) are significantly higher than the values for **PAH3** nanofibers (Ra = 0.81 nm, Rq = 1.71 nm) and **Lap** disks (Ra = 0.83 nm, Rq = 1.16 nm) due to the interactions between the two components **(**Figure 2e_i–iii**)**. We used transmission electron microscopy (TEM) and high-resolution TEM–energy dispersive spectroscopy (HRTEM-EDS) to characterize the coassembly. While TEM (Figure 2f_i–iii) shows the diameters of **PAH3** and **PAH3-Lap** nanofibers to be ∼9.8 nm and ∼12 nm, respectively, HRTEM-EDS elemental mapping shows colocalization of the characteristic element (N) on **PAH3** and the main elemental components (Si, Mg, and Na) of **Lap** (Supporting Information Figure S2). Such elemental colocalization is consistent with previous studies on Lap and silk coassembly.^37^

### Molecular Dynamics Simulations of PA-Lap Coassembly

#### Structural Optimization of PA–Nanoclay Interactions

In addition to the experimental evidence, we conducted molecular dynamics (MD) simulations (using Material Studio 8.0 molecular modeling package by Biovia)^51^ to further investigate the mechanism of PA-Lap coassembly. All MD simulations were conducted using the Forcite module with the COMPASS II (condensed-phase optimized molecular potentials for atomistic simulation studies) force field. The molecular structures of **PAH3** and controls **PAK3** and **PAE3** were built and optimized using the visualizer of Materials Studio 8.0. The structure of Lap is not available in the Material Studio 8.0 database; thus, we used Sepiolite (Mg_4_Si_6_O_15_(OH)_2_·6H_2_O) for this simulation due to its structural similarity with Lap. Like Lap, Sepiolite (**Sep**) is a layered hydrous magnesium silicate belonging to the 2:1 phyllosilicate family and made up of a 2D tetrahedral sheet of SiO_5_^4–^. While the slight structural differences between **Sep** and **Lap** may be the limit of this simulation, we believe the simulation provides insight into the dynamic interfacial interaction between PAs and layered inorganics. To investigate the interactions between PAs and **Sep**, first we built two kinds of cells: a small cell with two layers of clay (sepiolite) and one PA molecule and a second cell (Supercell) with enlarged layers with four or ten PA molecules. In this computational elucidation, we considered both electrostatic and van der Waals terms using atom-based summation methods with a repulsive cutoff of 12.5 Å. The energies of interaction (*E*_*inter*_) of **PAH3** with **Sep** in the cells with one, four, and ten **PAH3** molecules are −736.38, −3060.72, and −5626.32 kcal/mol, respectively (Supporting Information Table S2), thus suggesting that the total energy of the **PAH3-Sep** complex increases as further **PAH3** molecules are attracted to **Sep** to create higher-ordered nanostructures. In order to isolate the role of the imidazolium side chain of **PAH3**, we also investigated separately the interactions between a cationic **PAK3** and an anionic **PAE3** with **Sep** as controls. While interaction energy values for **PAK3-Sep** were negative, **PAE3-Sep** produces positive interaction energy. In both cases, these values increase with increasing number of molecules, suggesting that like **PAH3**, **PAK3** is attracted to the **Sep** surfaces while **PAE3** is strongly repelled.

#### Force Field MD Simulation Shows Stronger Interaction between **Lap** and **PAH3**

**Figure 3:**
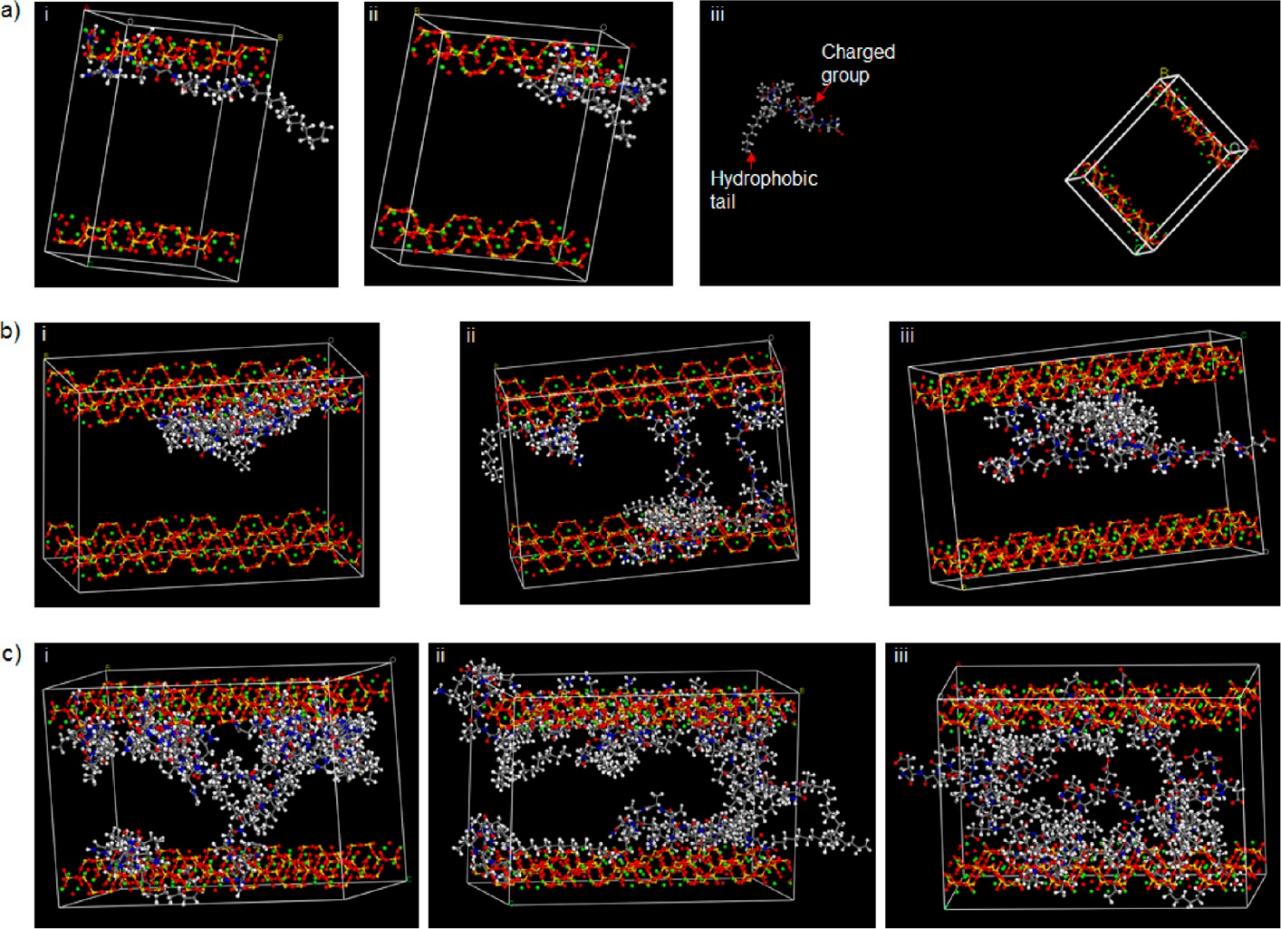
Molecular dynamics simulations of Sepiolite and PAs coassembly. (a) Layered Sepiolite cell with 1 molecule of (i) **PAH3**, (ii) **PAK3**, and (iii) **PAE3** after 1 ns dynamics steps. (b) Layered Sepiolite Supercell with 4 molecules of (i) **PAH3**, (ii) **PAK3**, and (iii) **PAE3** after 1 ns dynamics steps. (c) Layered Sepiolite Supercell with 10 molecules of (i) **PAH3**, (ii) **PAK3**, and (iii) **PAE3** after 1 ns dynamics steps.

Upon insertion of the PA molecules into supercells containing **Sep** nanoclay, we further confirmed the spatiotemporal orientation of the PA molecules within the lattice (26.80 × 53.60 × 37.64 Å) of the nanoclay. With one molecule in a small cell, **PAH3** and **PAK3** molecules were attracted to the nanoclay and oriented with their positively charged head groups in close proximity to the layer while the hydrophobic tails are displayed toward the space between the layers (Figure 3a_i–iii). As revealed in the supercells, the **PAH3** molecules accumulated on the surface of the nanoclay with the charged imidazolium side chain of the histidine residue facing the nanoclay surface (Figure 3b_i, c_i). In contrast, the **PAK3** molecules were more evenly distributed within the lattice (Figure 3b_ii, c_ii). The MD simulation also reveals that the negatively charged headgroups of **PAE3** molecules were facing toward the center of the supercells while the hydrophobic tails accumulated on the surface of the nanoclay (Figure 3b_iii, c_iii). Taken together, there was no observable interaction between the negatively charged **PAE3** and the nanoclay, whereas the cationic **PAH3** and **PAK3** preferentially interacted with the nanoclay surfaces through the charged head groups. However, **PAH3** displayed a much stronger affinity for the nanoclay to create a higher-ordered nanostructure, potentially attributable to an additional hydrogen bond contribution from the imidazolium side chain. These results demonstrate how by tuning the charged headgroup of PAs, it is possible to systematically optimize the supramolecular interactions between PAs and nanoclay nanomaterials, which will potentially determine the gelation kinetic and mechanical properties of the resulting PA–nanoclay hydrogels on a macroscale.

### Fabrication of PAH3-Lap Nanocomposite Hydrogels

**Figure 4:**
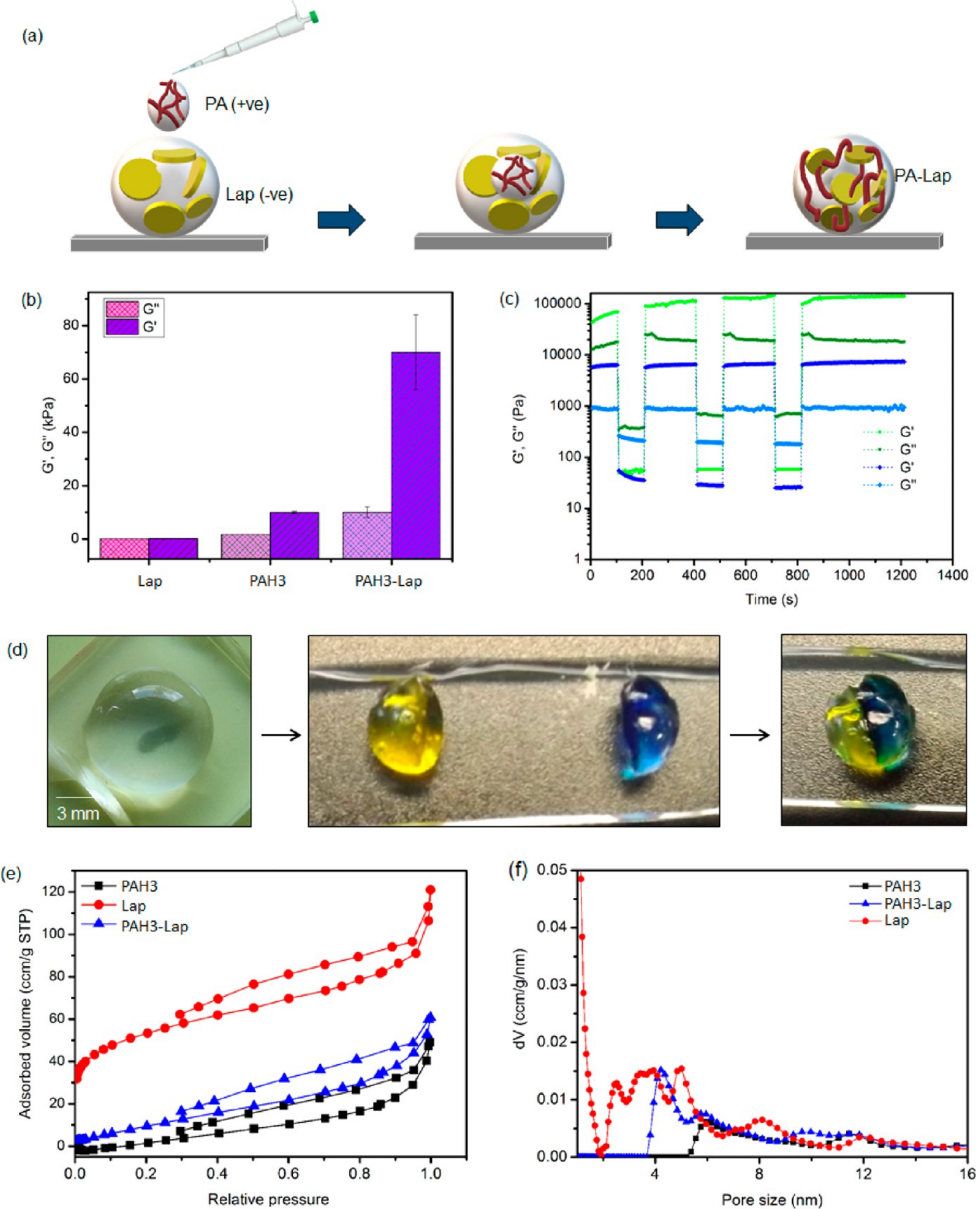
Surface and mechanical properties of hydrogels. (a) Schematic representation of **PAH3-Lap** hydrogel preparation. (b) Storage (*G*′) and loss (*G*″) moduli of **PAH3** and **Lap** compared to **PAH3-Lap** hydrogels. (c) Time sweep rheographs displaying thioxotropic properties of **PAH3** and **PAH3-Lap** hydrogels. (d) Optical image showing the robustness and self-healing capacity of **PAH3-Lap** hydrogels. (e) N_2_ sorption isotherms of **PAH3** (square traces), **Lap** (circular traces), and **PAH3-Lap** (triangular traces) xerogels. (f) Cumulative pore volume for **PAH3**, **Lap**, and **PAH3-Lap** xerogels.

Having established the underlying PA–nanoclay coassembling mechanism, we then focused on synthesizing hydrogels using **PAH3** and taking advantage of the modular nature of our material design. Given the unique chemistry of histidine, we prepared **PAH3-Lap** hydrogels by immersing **PAH3** solution (2% w/v) into a large volume of **Lap** solution (2.5% w/v) exfoliated with the sodium salt of poly(acrylic acid) (Mw = 5 kDa, 0.06% w/v). Within 30 min of immersion, self-supported hydrogels were formed in the **Lap** solution and the hydrogel was about the size of the **PAH3** droplet (∼10 mm), suggesting that the gelation was driven by a diffusion mechanism whereby **Lap** diffuses into the droplet of **PAH3** to trigger **PAH3-Lap** coassembly, which then leads to an entangled network of **PAH3** nanofibers and **Lap** nanodisks (Figure 4a). In contrast, immersion of **Lap** into **PAH3** did not produce stable hydrogels, which may result from a rapid diffusion of **PAH3** toward the **Lap** nanodisk suspension and inability to concentrate two components in a compartmentalized fashion. Also, when **Lap** was used without exfoliation, partial hydrogels were formed which might be attributed to electrostatic repulsion between the positive edges of **Lap** disks and the cationic imidazole side chain of **PAH3** or inhomogeneous dispersion of **Lap** in an aqueous medium. In contrast, the lysine-based analogue (**PAK3**; CH_3_-(CH_2_)_14_-CONH-VVVAAAKKK-CONH_2_) only formed weak hydrogels while the glutamic acid-based analogue (**PAE3**; CH_3_-(CH_2_)_14_-CONH-VVVAAAEEE-CONH_2_) did not cause gelation, suggesting that the presence of histidine aromatic imidazole head groups is key to preparing stable and strong hydrogels.

### Characterization of Mechanical and Surface Properties of PAH3-Lap Hydrogels

#### Application of Dynamic Rheometry to Characterize the Viscoelastic Properties of PA-Lap

In order to assess the impact of **Lap** on the mechanical properties of **PAH3** hydrogels, we used dynamic oscillatory rheology to measure the storage (*G*′) and loss (*G*″) moduli. *G*′ and *G*′′ of **PAH3-Lap** hydrogels were frequency independent, with *G*′ dominating *G*″ across the whole range of frequencies tested (0.1–50 Hz) and at constant strain γ (0.5%) (Supporting Information Figure S3). These results confirm a quasi-solid-like nature of **PAH3-Lap** hydrogels. The *G*′ (70.89 ± 10.62 kPa) and *G*″ (10.54 ± 2.11 kPa) values for **PAH3-Lap** nanocomposite hydrogels were greater than the *G*′ (10 ± 0.51 kPa) and *G*″ (1 ± 0.09 kPa) values of **PAH3** hydrogels (Figure 4b). To further confirm that supramolecular coassembly with **Lap** can improve the stiffness of other PA hydrogels, we prepared **PAK3-Lap** hydrogels. **PAK3** is known to produce weak hydrogels (*G*′ ∼ 1 kPa) by charge screening. Here, we observed that **PAK3-Lap** hydrogels displayed a *G*′ of ∼10 kPa (Supporting Information Figure S4), which is significantly lower than that of **PAH3-Lap** hydrogels (∼70.89 kPa). This enhanced stiffness of **PAH3-Lap** over **PAK3-Lap** is expected as the aromatic imidazole side chain of the histidine residue is known to play a critical role in promoting the self-assembly of proteinaceous fibers leading to self-healable and mechanically reinforced spider fangs, sandworm jaws, or mussel byssals.^47^ Therefore, we reasoned that the aromatic side chain of histidine might provide additional noncovalent interactions, making the surface free energy of adsorption (ε) of **PAH3** to **Lap** nanodisks greater or equal to the thermal energy (K_B_T).^52^ This is in agreement with our initial speculation based on the molecular dynamic simulations data (Figure 3).

In addition, we carried out strain amplitude sweep measurements to determine the strain-to-break values of **PAH3-Lap** against **PAH3**. The results indicated that *G*′ of **PAH3-Lap** decreased rapidly when subjected to a magnitude of strain beyond the critical strain value (γ = 6%). On the other hand, *G*′ values of **PAH3** hydrogels decreased rapidly at a much greater strain value (γ = 13%), suggesting that **PAH3** hydrogels are more viscoelastic than **PAH3-Lap** hydrogels. Put together, the enhanced stiffnesses of **PAH3-Lap** over **PAH3** and **PAK3-Lap** over **PAK3** suggest that the 2D structure of **Lap** promotes a strong physical interaction between the PA nanofibers and **Lap** nanodisks (Figure 1b). It is noteworthy that while PAs offer a powerful platform to design precise and bioactive matrixes, these materials tend to suffer from poor mechanical properties (*G*′ < 10 kPa), making our organic–inorganic hybridization an attractive strategy to prepare another class of PA-based hydrogels with dramatically improved mechanical properties (*G*′_PA-Lap_ ∼ 71 kPa). This strategy has been demonstrated using **Lap** with other organic components such as silk (*G*′_silk-Lap_ ∼ 150 kPa)^37^ and dendritic molecular binders (*G*′_dendron-Lap_ ∼ 250 kPa).^48^

#### Hydrogels Display Thixotropic and Self-Recovery Properties

Dynamic amplitude measurements were subsequently carried out to investigate the self-recovery or thixotropic property of **PAH3-Lap** and **PAH3** hydrogels following network rupture at high strain. We applied a high strain amplitude (100%) to rupture the hydrogel networks followed by a low strain amplitude (0.1%) to investigate the rate and extent of recovery of the hydrogels. Under the high strain amplitude (100%), the hydrogels underwent internal breakage leading to a significant decrease in *G*′ and inversion of *G*′ and *G*″. The inversion signifies that the liquid-like behavior dominates the solid-like nature of the hydrogels. When the strain amplitude was reduced to 0.1%, both **PAH3** and **PAH3-Lap** hydrogels displayed fast recovery within seconds (Figure 4c), making both types of hydrogels potentially injectable. While **PAH3** hydrogels exhibited complete recovery to the same initial *G*′, **PAH3-Lap** hydrogels exhibited enhanced recovery beyond the initial *G*′ (from 60 to 100 kPa) after the first strain cycle (Figure 4c_green trace). Such enhanced recovery has previously been reported in self-assembling hydrogels,^53,54^ and we reasoned it is suggestive of structural reorganization of the hydrogels. Macroscopically, **PAH3-Lap** hydrogels were able to self-heal in air (Figure 4d). These results suggest that **Lap** enhanced stability and facilitates self-healing in **PAH3-Lap** hydrogels. The rapid self-healing process exhibited by **PAH3-Lap** hydrogels may result largely from both the attachment of the imidazolium group of the **PAH3** to the exfoliated **Lap** surfaces and the intrinsic propensity of **PAH3** networks to rapidly recover after rupture.

### Characterization of Surface Properties of PAH3-Lap Hydrogels

Having characterized the bulk properties of **PAH3-Lap** hydrogels, we then used a nitrogen gas adsorption method based on quenched solid density functional theory (QSDFT) to investigate the impact of PA-Lap coassembly on surface properties including pore volumes and pore diameters. The experiments were conducted on **PAH3-Lap** dried xerogels and compared to the individual components. In all cases, the xerogels exhibited a surface profile that is consistent with type-III adsorption–desorption curves with distinct capillary condensation steps. The adsorption isotherms (volume of nitrogen per gram of materials at standard temperature and pressure (STP)) revealed that the surface areas of **Lap** and **PAH3** xerogels were 165 m^2^/g and 18 m^2^/g, respectively (Figure 4e, Supporting Information Figure S5). Upon coassembly, the surface area (50 m^2^/g) of **PAH3-Lap** was higher than that of **PAH3** xerogels, implying that the surface adsorption of **PAH3** to **Lap** nanodisks considerably decreases the surface area of the **Lap** nanodisks. For **Lap**, a number of peaks which span between 0.25–0.78 nm (micropores), 2.00–6.00 nm (mesopores), and 12.00 nm (macropores) (Figure 4f_red trace) were observed, suggesting that **Lap** displays a hierarchical polymodal pore size distributions.^55,56^ In contrast, **PAH3** xerogel pore size distribution profiles displayed a broad peak centered at 6 nm (mesopores) and another weak peak at 12 nm, indicating that **PAH3** xerogels exhibited a uniform pore size distribution (Figure 4f_black trace). It is important to note that the observed porosity profile for **PAH3** xerogels could be due to lyophilization of the gels prior to analysis. The pore size distribution curves for **PAH3-Lap** showed multiple peaks centered at 4.2, 6.0, 10.0, and 12.0 nm. The peaks at 4.2 and 6.0 nm are characteristic fingerprints of **Lap** and **PAH3** xerogels, respectively (Figure 4f_blue trace), which confirm the heterogeneity of the **PAH3-Lap** internal structure. In contrast, the peaks corresponding to the micropores of **Lap** are not apparent in **PAH3-Lap**, which suggests that surface adsorption of the **PAH3** nanofibers to the **Lap** disk removes access to the micropores by blocking them. We therefore hypothesized that the molecular diversity and heterogeneous functional groups of **PAH3-Lap** hydrogels in relation to **PAH3** hydrogels may provide an opportunity to nucleate and grow apatite crystals within the confined 3D framework of the hydrogels.

### PAH3-Lap Hydrogels to Guide *In Situ* Mineralization

#### Features That Make Organic–Inorganic Hydrogels an Ideal Model for Biomineralization

Hydrogels have been harnessed as structural frameworks to elucidate the origins of biological control over crystal morphology, orientation, and matrix incorporation.^3^ These materials display (i) volumetric confinement to control crystal growth, (ii) nanoporosity to control diffusion rates, capacity to tune concentrations and supersaturation of solutes, and (iii) internal nanostructures with high surface area to template crystal growth.^3,57^ Unlike the classical mechanism of atom or molecule mediated growth of single crystals, the particle mediated growth and assembly mechanisms leading to the formation of single crystals have been recognized as emerging nonclassical biomineralization processes.^58^ This phenomenon is believed to result from both the free-energy landscapes and reaction dynamics that govern particle–particle interactions.^59−61^ Such reaction dynamics might account for the impressive mineral deposition recently observed by Paul and co-workers using DNA–Laponite hybrid hydrogel coatings on bone allografts.^36^ Therefore, we hypothesized that the surface area, functional groups, and structural anisotropy of **Lap** nanodisks can be harnessed in **PAH3-Lap** hydrogels to control the energy landscape at the substrate–nuclei interface during biomineralization in a time-dependent manner, leading to the formation of multi-inorganic–organic nano-objects.

**Figure 5:**
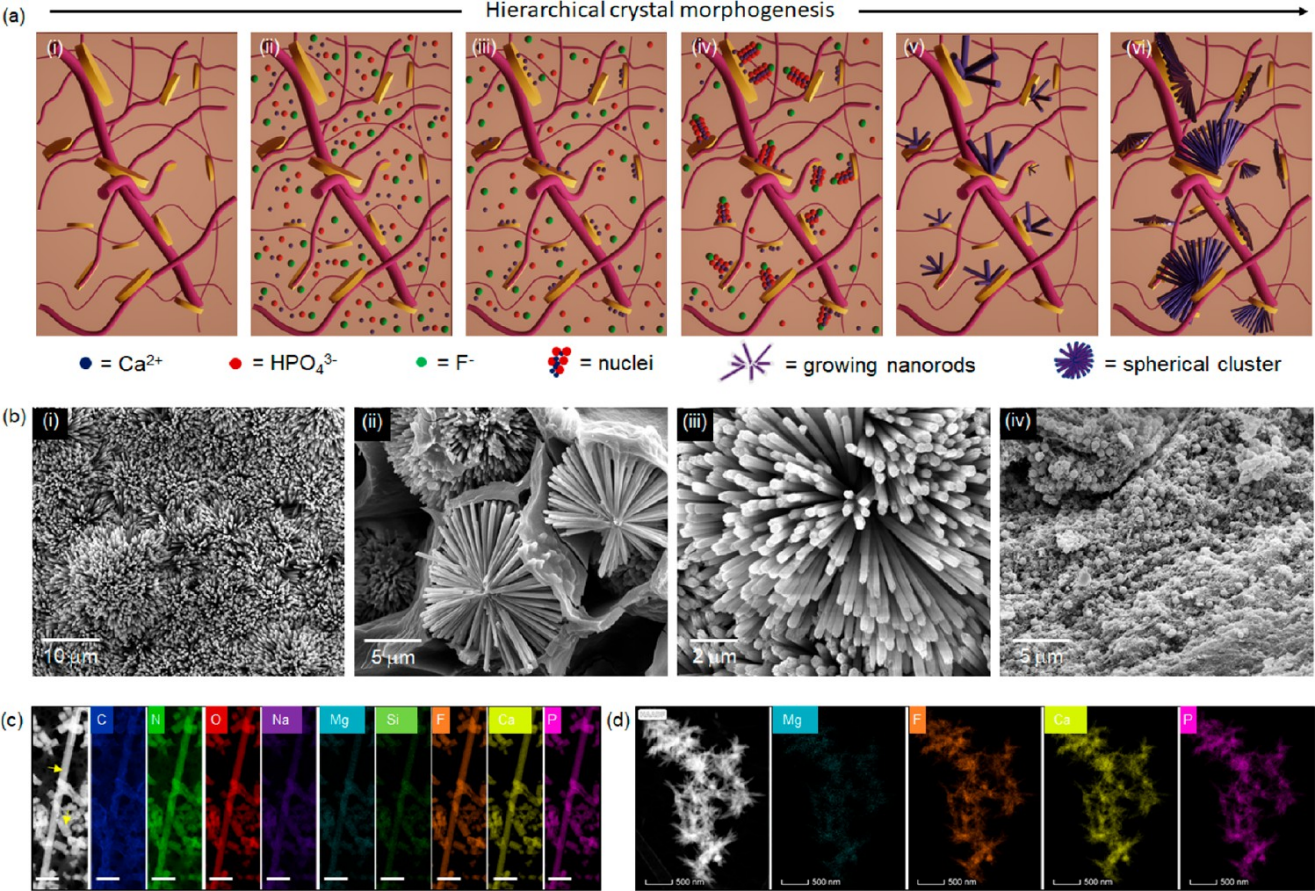
Biomineralization in hydrogels. (a) Schematic representation of the biomineralization process. **PAH3-Lap** hydrogel was immersed in a mineralizing bath (i) followed by a gradual diffusion of the ionic precursors into the hydrogel cavity (ii), which resulted in an initial electrostatic binding of calcium (Ca^2+^) ions to the **Lap** surface (iii). Further association of phosphate (HPO_4_^3–^) and fluoride (F^–^) ions with the Lap-Ca^2+^ complex produces nuclei (iv) that developed into oriented nanorods in a time-dependent manner (v), which organize hierarchically into spherical clusters (vi). (b) Scanning electron micrographs showing (i) dense crystal formation on the surface of a **PAH3-Lap** hydrogel, which are organized into (ii, iii) spherical clusters of nanorods within the cavity of the hydrogels after 8 days in mineralizing solution. Also, **PAH3** hydrogels were mineralized but spherical and amorphous crystals were formed (iv). (c) HRTEM-EDX elemental mapping of nanorods formed in **PAH3-Lap** hydrogels after 8 days. Yellow arrows indicate fluoridated hydroxyapatite nanorods. Elemental mapping of the nanorods shows carbon C (blue), nitrogen N (green), oxygen O (red), sodium Na (purple), magnesium Mg (cyan), silicon Si (green), fluorine F (orange), calcium Ca (yellow), and phosphorus P (pink). Scale bar: 100 nm. This was contrasted with the morphology of the (d) needle-like crystals that formed in **PAH3-Lap** hydrogels within 2 h of incubation in mineralizing solutions.

To test this hypothesis, we used a known mineralizing solution to nucleate and trigger the growth of fluoridated hydroxyapatite nanocrystals.^10^ The **PAH3-Lap** hydrogels were submerged in the mineralizing solution (20 mL) and kept at 37 °C (Figure 5a). We observed that the transparent **PAH3-Lap** hydrogels became cloudy within 8 days of incubation (Supporting Information Figure S6), suggesting its high mineralization capacity. In contrast, **PAH3** hydrogels remained less opaque, indicating less mineralization than **PAH3-Lap** hydrogels. In order to confirm that this enhanced mineralization was due to the presence of **Lap**, we added **Lap** solution alone to the mineralizing solution and investigated crystal formation. Interestingly, **Lap** suspension exhibited white precipitates after 8 days of incubation, suggesting that **Lap** is able to drive nucleation and growth of apatite crystals. These results are potentially consistent with the ability of **Lap** nanodisks to act as catalysts for the formation of mineralized matrixes both in *in vitro* and *in vivo*.^32−34^ Similarly, silica hydrogels have previously been used to drive the formation of hematite (αFe_2_O_3_) into hierarchical mosaic crystals displaying hierarchical structures inaccessible in solution-grown controls, indicating that silicate materials display functionalities that promote heterogeneous nucleation and growth of crystals.^62^

#### Lap Nanodisks Are Essential for Nanorod Formation

Scanning electron microscopy (SEM) was used to examine the mineralization within the hydrogels. SEM micrographs of **PAH3-Lap** xerogels revealed the presence of high-aspect ratio apatite nanorod crystals (∼50 nm in cross-sectional diameter) on the surface of the mineralized hydrogels after 8 days in the mineralizing solution (Figure 5b_i). These apatite nanorods were organized hierarchically into well-defined microscopic clusters, which resemble mesocrystals.^63^ The clusters grew symmetrically and to similar sizes up to microns in diameter in “confined pockets” within the hydrogels (Figure 5b_ii–iii). We hypothesize that these cluster structures are formed by the diffusion of ionic mineralization precursors through the **PAH3-Lap** hydrogel, random nucleation across the internal walls of the hydrogel, and subsequent symmetric growth of apatite nanorods along the precursor–crystal interface (Figure 5c). This process of crystal growth and entrapment within integrated hybrid materials has been regarded as nanoscale incarceration by Mann.^17^ In contrast to this nanorod and microcluster organization within **PAH3-Lap** hydrogels, we observed spherical nanocrystals (diameter ∼50 nm) in **PAH3** hydrogels, which are reminiscent of previous studies by Stupp and colleagues.^64^ Based on these results, we propose that the integrated nanofibers and nanodisks within **PAH3-Lap** hydrogels provide a 3D organic–inorganic framework of heterogeneous nucleation sites for hierarchical mineralization. To explore the possibility that **Lap** is acting as a catalyst for mineralization in the **PAH3-Lap** hydrogels, we hybridized **Lap** with **PAK3** knowing that **PAK3** does not induce mineralization of apatite in its own right. In this case, we again observed formation of both nanorods and nanospheres within the hydrogels after 8 days of incubation (Supporting Information Figure S7), thus suggesting that the presence of **Lap** nanodisks in PA-based hydrogels played a key role in the nucleation and growth of crystals within the hydrogels.

#### Elemental Mapping to Elucidate Colocalization of **Lap** and Hydroxyapatite

Given the hierarchical nanorod-cluster mineralization within **PAH3-Lap**, we then investigated nanorod crystal formation in further detail. First, to verify interactions between **Lap** nanodisks and the mineralization ionic precursors, we used HRTEM-EDS to map the elemental composition of the mineralized **PAH3-Lap** hydrogels. HRTEM images confirmed the formation of the ∼50 nm diameter hexagonal nanorod crystals in the **PAH3-Lap** hydrogels after 8 days of incubation (Figure 5c). Also, the HRTEM-EDS mapping revealed colocalization of carbon (C), nitrogen (N), oxygen (O), sodium (Na), magnesium (Mg), silicon (Si), fluoride (F), calcium (Ca), and phosphorus (P) along the nanorods, which suggests the incorporation of dissolved **PAH3** and **Lap** into the nanocrystals during growth. To gain insight into the early stage of this mineralization phenomenon, we examined the morphology and elemental composition of the crystals obtained after a 2 h incubation period in the mineralizing solution. HRTEM-EDS micrographs of **PAH3-Lap** hydrogels following a 2 h incubation period revealed an outward growth of the spherical clusters comprising the nanorods with colocalized elemental components of **PAH3-Lap** hydrogels (Figure 5d). Moreover, the nanorods appeared to be growing in the direction of the **PAH3-Lap** hydrogel nanofibers (Supporting Information Figure S8a), which suggests that the orientation of the nanofiber–nanodisk hybrid might be playing a key role in directing the hierarchical nanorod growth. Also, the white particles that sediment in the **Lap** solution were analyzed using HRTEM-EDS, which revealed the formation of agglomerated nanorods with elemental mapping showing both **Lap** characteristic elements and ionic precursors for mineralization (Supporting Information Figure S9). These results indicate that **Lap** might be serving as an essential template for nanorod growth within the organic–inorganic hydrogels due to its 2D ultrathin structure and surface chemistry.

#### FTIR Confirms Hydrogen Bond-Driven Interactions between **Lap** and Biominerals

**Figure 6:**
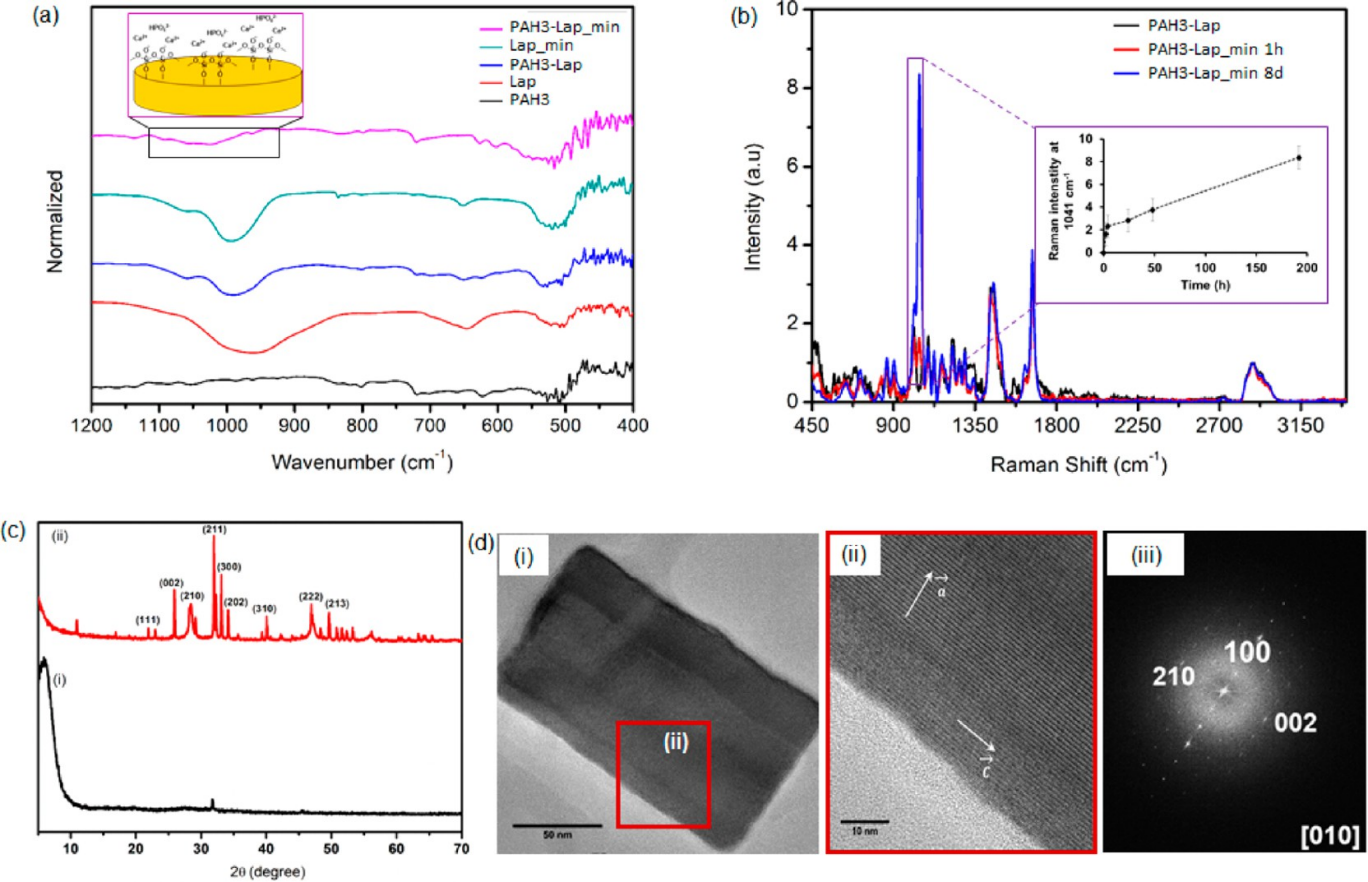
Characterization of biomineralization. (a) FTIR spectra indicating **Lap** silica oxide layer participation in biomineralization. (b) Normalized Raman spectra of unmineralized **PAH3-Lap** hydrogels and **PAH3-Lap** mineralized for 1 and 8 days. The inset is a plot of 1047 cm^–1^ signal intensity versus mineralization time. (c) X-ray powder diffraction (XRD) crystallographic profiles of mineralized (red trace) and unmineralized (black trace) **PAH3-Lap** hydrogels after 8 days of incubation in mineralizing solution. (d) (i) HRTEM image of hydroxyapatite nanorods formed in **PAH3-Lap** hydrogels, (ii) enlarged selected area (red square) of HRTEM image of nanorods, and (iii) FFT patterns of crystals viewed from the [010] crystallographic direction.

Using Fourier transform infrared (FTIR) spectroscopy, we then investigated the mechanism of interaction between **Lap** and the ionic precursors present in the mineralizing solution. With the technique we also attempted to verify the identity of the apatite nanorods. According to the FTIR spectra (Figure 6a), the band at 970 cm^–1^ corresponds to Si–O–Si of **Lap**. This band shifts from 970 to 985 cm^–1^ in **PAH3-Lap** hydrogels, which suggests hydrogen-bonding interactions between **PAH3** and **Lap**. Such red-shift in the Si–O–Si band of **Lap** has previously been observed in polymer–Lap composite hydrogels.^65^ After incubating **PAH3-Lap** in the mineralizing media for 8 days, the band became broader and was further shifted to a higher frequency (*ca*. 1022 cm^–1^). Thus, we hypothesized that the mineralized **PAH3-Lap** hydrogels interacted noncovalently with the Si–OH layer of **Lap**. To verify this, we incubated a **Lap** suspension in the mineralizing media under the same conditions for 8 days. The band of Si–OH shifted from 970 to 995 cm^–1^, confirming **Lap** as an active catalyst for mineralization in **PAH3-Lap** hydrogels.

#### Time-Resolved Evolution of Nanocrystals and Associated Fingerprints to Understand Mechanisms of Biomineralization

Given the distinctive functional groups of **Lap, PAH3**, and the mineralized nanorods, Raman spectroscopy was used to elucidate their molecular composition within mineralized **PAH3-Lap** hydrogels. Furthermore, by taking advantage of the fingerprints of phosphate functional groups on the nanorods, we monitored the kinetics of crystal growth in **PAH3-Lap** hydrogels. The Raman spectra of the **PAH3-Lap** hydrogels mineralized for 8 days revealed vibrational frequencies corresponding to the internal PO_4_^3–^ mode. The vibrational frequencies of the PO_4_^3–^ were found to be ν1 = 960 cm^–1^ and ν3 = 1047 cm^–1^ (Figure 6b). These frequencies correspond to the characteristic symmetric P–O stretching modes and the triply degenerate asymmetric P–O stretching modes, respectively.^66^ Peaks at 1450 cm^–1^ (C=C stretch of the imidazole side chain) and 1675 cm^–1^ (C=O stretch, amide band I) correspond to peptide vibrations from **PAH3** while the peak at 1010 cm^–1^ corresponds to the Si–O vibrational stretch from **Lap** present in the **PAH3-Lap** hydrogels. The amide band I at 1675 cm^–1^ further confirms the intrinsic β-sheet conformation of **PAH3** nanofibers in the coassembled **PAH3-Lap**.^67^ By comparing this amide band I before and after mineralization, we observed no significant changes in the conformation of **PAH3** nanofibers, thus suggesting that the **PAH3** nanofibers maintained their spatial organization under the mineralization event.

We monitored the kinetics of crystal growth in the **PAH3-Lap** hydrogels by observing the regions of the Raman spectra corresponding to the triply degenerate asymmetric P–O stretching modes (ν3 = 1047 cm^–1^). At time *t = 0* (before mineralization), no Raman peak was apparent in this region. After mineralization for 1 h, there was an emergence of the P–O stretching mode that featured two sharp peaks at 1005 and 1047 cm^–1^ (Figure 6b, Supporting Information Figure S10). The relative intensity of the 1047 cm^–1^ peak signal (all spectra were first normalized with respect to the C–H signal intensity at 2800–3000 cm^–1^) increased rapidly within 4 h of mineralization and steadily afterward until the 8-day time-point (Figure 6b_inset, Supporting Information Figure S10), indicating a two-phase crystal growth. Elemental analysis of the two stages of crystal growth revealed a Ca/P ratio of 1.45 and 1.65 for the nanorods obtained at 4-h and 8-day time-points, respectively (Supporting Information Figure S8d). The former Ca/P ratio is indicative of an amorphous calcium phosphate while the latter is characteristic of a hydroxyapatite crystal. Thus, the two-stage crystallization events exhibited an initial amorphous precursor phase, which steadily underwent a slow interaction with the mineralization ionic species diffused into the **PAH3-Lap** hydrogels, leading to a linear rate of growth to attain the hydroxyapatite composition with Ca/P ratio 1.65. Similarly, Raman spectra for **PAH3** hydrogels mineralized for 8 days also displayed the key PO_4_^3–^ fingerprints (ν1 = 960 cm^–1^, ν3 = 1047 cm^–1^) of hydroxyapatite formed in **PAH3-Lap** hydrogels as well as a Raman peak at 564 cm^–1^ (Figure 6b), which corresponds to the ν4 bending mode characteristic of PO_4_^3–^ in amorphous calcium phosphate.^67^ We also used elemental analysis to show that the Ca/P ratio is 1.1 for the amorphous calcium phosphate formed in the **PAH3** hydrogels (Supporting Information Figure S12).

#### XRD and Other Physical Analysis Techniques Confirm the Crystallographic Direction of Crystal Growth in the **PAH3-Lap** Hydrogels

The X-ray diffraction pattern of the mineralized **PAH3-Lap** hydrogels compared to the unmineralized **PAH3-Lap** hydrogels indicated that the nanocrystals formed after 8 days were crystalline (Figure 6c). More so, the diffraction peaks (002) at 2θ = 25.8°, (211) at 2θ = 31.8°, (300) at 2θ = 32.8°, (202) at 2θ = 34.2°, and (222) at 2θ = 46.9° (Figure 6c_ii) are consistent with the peaks for fluoridate hydroxyapatite.^57^ Furthermore, the sharp 002 peak indicated that the nanorods were oriented along the c axes, which is reminiscent of nanocrystal growth in both dental enamel and bone.^68^ A closer look at the HRTEM images of the mineralized **PAH3-Lap** hydrogels confirmed that the nanorods assumed a preferred orientation along the c axes of the fluoridated hydroxyapatite (Figure 6d_i–ii). Also, the fast Fourier transform (FFT) patterns viewed from the [010] crystallographic direction are consistent with the XRD data and HRTEM images, indicating that the crystal lattices were only observed in the nanorods with long axes along the direction corresponding to the reflection area (Figure 6d_iii). These results further confirm that the c axes of the crystal lattices were mainly aligned along the long axes of the nanorods. Although, the potential of **Lap** to trigger cascades of cell signaling that mediate bone formation *in vivo* is well-known,^28^ these results showcase the potential of **Lap** nanodisks as efficient templates to guide nanocrystal growth via a nonclassical particle attachment mechanism and in a hierarchical manner.

### Evaluation of Biocompatibility of Mineralized PAH3-Lap Hydrogels

**Figure 7:**
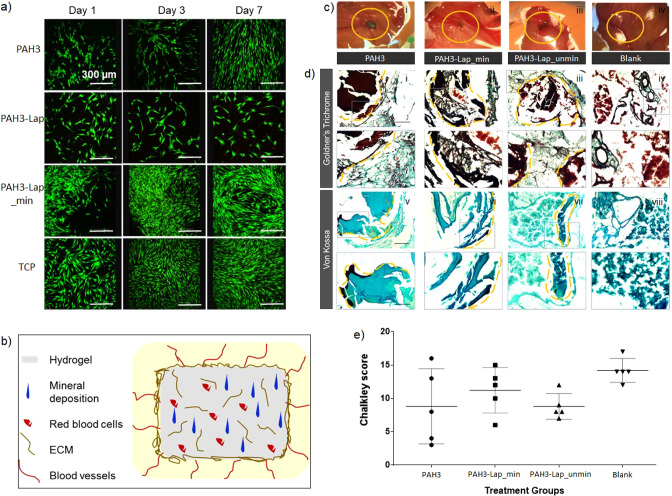
Biological applicability of PAH3-Lap hydrogels. (a) The *in vitro* applicability of the hydrogels was assessed by a LIVE/DEAD assay to test cell viability and proliferation of hBMSC on the hydrogels. The results revealed that cell viability and proliferation on mineralized **PAH3-Lap** hydrogels are more than those of cells growing on tissue culture plastic (TCP) for 7 days. (b) Schematics of CAM implantation of hydrogels. Hydrogels promote mineral deposition, red blood cell infiltration, and ECM and blood vessels formation. (c) Optical image of CAM implanted hydrogels. (i) **PAH3** hydrogels implanted in CAM were surrounded by blood vessels in close proximity of the chorioallantoic membrane. Both (ii) **PAH3-Lap** and (iii) **PAH3-Lap**_**-**_**min** hydrogels were highly integrated with the chorioallatoic membrane with blood vessels penetrating the implanted hydrogels. (d) Histological analysis of CAM implanted hydrogels. Mineral deposition was found limited to the outer region of the implanted **PAH3** hydrogels. Limited mineral deposition was observed in proximity of the **PAH3-Lap** hydrogel–membrane interface. Mineral deposition was found extensively within the **PAH3-Lap**_**-**_**min** hydrogels. Both **PAH3-Lap** and **PAH3-Lap**_**-**_**min** hydrogels were extensively invaded by red blood cells. Blank samples were extensively penetrated by blood vessels but no mineral deposition. (e) Chalkley score of **PAH3**, **PAH3-Lap**, **PAH3-Lap_min**, and blank controls samples. Statistical significances were assessed by one-way ANOVA. Mean ± SD *n* = 5. Scale bar for (d) = 100 μm.

The biological relevance of **PAH3** as well as mineralized and unmineralized **PAH3-Lap** hydrogels as functional biomaterials was assessed *in vitro* by seeding human bone marrow stromal cells (hBMSCs) on the hydrogels. As shown in Figure 7a, live skeletal cells stained with calcein AM were predominantly visible on the hydrogels after 7 days in culture, indicating excellent cytocompatibility across the hydrogels. However, hBMSCs proliferated significantly more on the mineralized **PAH3-Lap** hydrogels compared to the **PAH3** and unmineralized **PAH3-Lap** hydrogels, as well as on tissue culture plastic (TCP) (Supporting Information Figure S13). To further assess the biological functionality of the hydrogels *ex vivo*, we used the chorioallantoic membrane (CAM) assay of the chick embryo, to examine tissue integration and blood vessel and extracellular matrix formation as previously published.^69^ Histological analysis of the implanted **PAH3** and unmineralized and mineralized **PAH3-Lap** hydrogels after 7 days demonstrated that the hydrogels fully integrated within the CAM (Figure 7c_ii). However, while blood vessels were only visible on the surface of the **PAH3** hydrogels (Figure 7c_i), both unmineralized (Figure 7c_iii) and mineralized (Figure 7c_ii) **PAH3-Lap** hydrogels exhibited blood vessels growing within (Figure 7c_ii–iii), indicating a higher capacity of neovascularization. Using Goldner’s and von Kossa staining, we confirmed extensive mineral deposition in the mineralized **PAH3-Lap** (**PAH3-Lap**_**-**_**min**) hydrogels (Figure 7d_ii,vi) in comparison to unmineralized hydrogels (Figure 7c_iii,vii). No mineral deposition was apparent in the blank eggs (Figure 7d_iv,viii). Similar to the blank samples, both **PAH3-Lap** (Figure 7d_iii) and **PAH3-Lap-min** (Figure 7d_ii) hydrogels were extensively invaded by red blood cells. The Chalkley score (Figure 7e) shows there is no significant difference between the level of vascularization in the treatment groups and the controls. These results suggest that the **PAH3-Lap** hydrogels can serve as robust multifunctional matrices with the capacity to promote cell growth, trigger hierarchical mineralization and bone tissue formation, and promote vascularization.

## Conclusion

We have developed a coassembling organic–inorganic hydrogel platform for *in vitro* crystal growth mediated by a particle attachment mechanism within a 3D supramolecular confined framework. The design strategy hinges on electrostatic interactions between **Lap** nanodisks and cationic **PAH3** molecules to integrate the intrinsic properties of the organic and inorganic components into distinctive organic–inorganic hydrogel structures. The resulting materials displayed high surface area, high mechanical properties, and self-healing properties. Furthermore, the coassembling **PA-Lap** hydrogel displayed a nanoscale architecture that served as confined spaces for the hierarchical growth of hydroxyapatite from ordered nanorods into well-defined spherical clusters. The study explores this mineralization mechanism as a biomimetic 3D model to modulate nucleation and spatiotemporal organization of fluoridated hydroxyapatite. This model was used to understand the role of both **Lap** nanodisks and **PAH3** nanofibers within **PAH3-Lap** hydrogels in guiding the growth of the hydroxyapatite nanorods across multiple length scales. At the atomic level, the mineralization of **PAH3-Lap** depended on a diffusion-driven process where local ionic concentration and supersaturation are mediated by supramolecular interactions with **Lap**. Furthermore, the nanoscale architecture of the **PAH3-Lap** hydrogels facilitated incarceration of the nanorod crystals and subsequent growth into the distinctive spherical clusters at the microscale. Interestingly, these mineralized **PAH3-Lap** nanocomposite hydrogels outperformed all control groups in supporting cell growth, stimulation of cell ingress, blood vessel infiltration, ECM production, and mineral deposition in a CAM model. In addition to these advantages, the shear-thinning property of the system makes it a suitable material to serve as a bioink for 3D printing applications. Overall, this study presents a nanotechnology approach to the design of integrated and higher-ordered self-assembling nanomaterials with potential widespread applications in regenerative medicine.

## Experimental Methods

### Zeta Potential (ζ)

All ζ-potential measurements were performed after resuspension of the PAs at a concentration of 0.1% w/v in ultrapure water. After loading the samples into folded capillary cells, measurements were performed at 25 °C using a ζ-sizer instrument (Nano-ZS Zen 3600, Malvern Instruments, UK). For each PA, three separate samples were measured with at least five runs per sample.

### Circular Dichroism Spectroscopy

Circular dichroism (CD) was measured with a Chirascan circular dichroism spectrometer (Applied Photophysics Limited, UK) using a quartz cell with a 1 mm path length and the following parameters: data pitch, 0.5 nm; scanning mode, continuous; scanning speed, 100 nm/min; bandwidth, 2 nm; accumulation, 5. All CD data are presented as ellipticity and recorded in millidegree (mdeg). CD measurements were performed on aqueous solutions of **PAH3** (0.1% w/v), **Lap** (0.25%), and their mixtures. CD spectra were obtained by signal integrating 3 scans, from 190 to 260 nm at a speed of 50 nm/min. Data were processed by a simple moving average and smoothing method.

### Small-Angle Neutron Scattering (SANS) Analysis of Hydrogel Nanostructures

Synchrotron small-angle neutron scattering (SANS) measurements were performed on the fixed-geometry, time-of-flight LOQ diffractometer (ISIS Neutron and Muon Source, Oxfordshire, UK). A white beam of radiation with neutron wavelengths spanning 2.2 to 10 Å enabled access to a *Q* [*Q* = 4π sin(*θ/2*)/λ] range of 0.004 to 0.4 Å^–1^ with a fixed-sample detector distance of 4.1 m. The cuvettes were mounted in aluminum holders. The time taken for each measurement was approximately 30 min. All scattering data were normalized for the sample transmission, the backgrounds were corrected using a quartz cell filled with D_2_O, and the linearity and efficiency of the detector response were corrected using the instrument-specific software.

### Atomic Force Microscopy (AFM)

AFM was performed on a Bruker Multimode 8 AFM with a Nanoscope V controller using PeakForce Tapping mode with a ScanAsyst Air cantilever (spring constant 0.4 N/m). The cantilever was calibrated using the automated “no touch” calibration routine built into the software. Solutions of **PAH3** (0.01% w/v, 40 μL), **Lap** (0.025% w/v, 40 μL), and **PAH3/Lap** mixtures were dropped onto freshly cleaved mica surfaces. The samples were air-dried at room temperature for 24 h and imaged with a PeakForce set point of 500 pN with a PeakForce amplitude of 30 nm and frequency of 4 kHz. Images were acquired at 512 × 512 pixels at a line rate of 2.8 Hz. The height images were processed in the Nanoscope Analysis software after using first order flattening to remove tilt. Images were processed in Nanoscope 1.7.

### Transmission Electron Microscopy (TEM) and High-Resolution TEM (HRTEM)

Aqueous solutions of **PAH3** (0.01% w/v) and Lap (0.025% w/v, exfoliated with 0.0068% w/v ASAP) were dissolved in ultrapure water. Similarly, mixtures of **PAH3** (0.02% w/v) and **Lap** (0.5 wt %/v) were also prepared. Samples were mounted on a copper TEM plasma etched holey carbon-coated copper grid (Agar Scientific, Stansted, UK). The grids were immersed in the sample solutions for 5 min. Excess was removed on filter paper before incubation with 2% uranylacetate solution for 30 s. Grids were then washed with ultrapure water for 30 s and air-dried for 24 h at room temperature. Bright-field TEM imaging was performed on a JEOL 1230 transmission electron microscope operated at an acceleration voltage of 80 kV. All the images were recorded by a Morada CCD camera (Image Systems). At least three images were taken per sample for further analysis. High-resolution transmission electron microscope (HRTEM) images, selected area electron diffraction (SAED) patterns, scanning transmission electron microscope (STEM) images, and energy dispersive X-ray spectroscopy (EDS) spectrum images were obtained with a FEI Talos F200X microscope equipped with an X-FEG electron source and Super-X SDD EDS detectors. The experiment was performed using an acceleration voltage of 200 kV and a beam current of approximately 1 nA. TEM images were recorded with a FEI CETA 4k x 4k CMOS camera. STEM images were acquired with HAADF and BF detectors.

### Preparation of Hydrogels

An aqueous solution of **Lap** (2.5% w/v) was prepared by adding the requisite amount of Lap powder to a stirred suspension of ASAP (0.06% w/v) in ultrapure water. The **Lap** suspension was sonicated for 30 min until a clear transparent sample was obtained. Aqueous solutions of **PA** (2% w/v) were prepared in HEPES buffer. **PA-Lap** hydrogels were prepared by injecting a solution of **PA** (20 μL) into a larger volume of **Lap** (100 μL). Gelation was allowed to proceed overnight at room temperature. Hydrogels of **PAH3** (2% w/v) were prepared by basifying an aqueous solution of **PAH3** with NaOH (1 M).

### Dynamic Rheological Measurements

Rheological measurements were performed using a Discovery Hybrid Rheometer, Rheo-DHR3 (TA Instruments). All data were collected at 25 °C. The preformed hydrogels were added to the center of the bottom plate, and the top parallel plate (with 8 mm diameter) was lowered to a gap of 100 μm. The amplitude sweep measurements were performed between 0.1 and 50% strain at constant frequency (1 Hz). Similarly, frequency sweep rheographs were obtained between 0.1 and 20 Hz at constant strain (0.5%). Self-healing was assessed initially at 0.1% strain for 100 s, then at 100% strain for 200 s, 0.1% strain for 200 s, 100% strain for 200 s, and 0.1% strain for 400 s.

### Characterization of Surface Properties of Xerogels

Nitrogen sorption isotherms of the lyophilized xerogels were measured at 77 K using an Autosorb-IQ system (Quantachrome Instrument, USA). Before measurements, the samples were degassed in a vacuum at 120 °C overnight. The specific surface areas (*S*_BET_) were calculated by the multipoint Brunauer–Emmet–Teller method using adsorption data in a relative pressure range from 0.04 to 0.2, and the pore-size distribution was calculated based on quenched solid density function theory (QSDFT) using the adsorption branches of isotherms assuming slit and cylindrical pore geometries. By using the Barrett–Joyner–Halenda (BJH) model, the mesoporous surface areas (S_BJH_) were calculated from the adsorption line. The microporous surface areas (S_DR_) were calculated from the adsorption line by the Dubinin–Radushkevich (DR) model.

### Biomineralization of Hydrogels

The mineralizing solutions were prepared as previously reported by Elsharkawy et al.^10^ Briefly, an aqueous suspension of hydroxyapatite powder (2 mM) and sodium fluoride (2 mM) was prepared in deionized water with continuous stirring. Then, 69% nitric acid was added dropwise to the suspension to aid a complete dissolution of the hydroxyapatite precipitates at pH 2.4. Thereafter, an aqueous solution of ammonium hydroxide (30%) was added dropwise to the hydroxyapatite solution until it reached pH 6. Various hydrogels were then immersed in the hydroxyapatite solutions and incubated for 8 days at 37 °C using a temperature-controlled incubator (LTE Scientific, Oldham, UK).

### Monitoring of the Biomineralization Process by Raman Spectroscopy

All Raman analysis was carried out on a confocal WITEC Alpha300 system utilizing a 785 nm laser and a 20× (S Plan Fluor, NA 0.45, ELWD) objective lens. Raman scatter was collected in a backscattering geometry. A small amount of each sample was placed on a microscope glass slide which had been previously cleaned with a methanol-soaked tissue, with a new slide used for each sample. The incident laser power was constant for all samples at 63 mW. No signal loss was observed, for example due to photobleaching or carbonization, when samples were irradiated on the same spot in triplicate with integration times ranging from 10 to 60 s. All spectra processing was performed using SpectraGryph 1.2 involving (1) cosmic ray removal, (2) background correction, and then (3) subsequent normalization. An advanced baseline correction protocol available in the SpectroGryph software was applied which fits a polynomial curve to the spectral regions where there is no Raman peak and enables subtraction of the variable y-offset associated with the luminescence background. To enable comparison of the relative changes in the Raman intensity of the 1047 cm^–1^ peak in Figures 6 and S10, all spectra were normalized with respect to the peak intensity in the 2800–3000 cm^–1^ region. This approach was adopted as the integration time was varied between samples to optimize the signal-to-noise ratio alongside variation in background luminescence with mineralization times. However, the C–H vibrational spectral shape across 2800–3000 cm^–1^ remained relatively unchanged for each sample, and the Raman peak intensity was also observed to change proportionally with integration time in this region. For each measurement, multiple spectra were acquired across the sample with the focus depth also optimized, which revealed good uniformity and ensured that the spectra presented are representative of the sample.

### Synthesis and Purification of Peptide Amphiphiles

The peptide amphiphiles (PAs) were synthesized using solid-phase peptide synthesis (SPPS) on a Liberty Blue automated microwave peptide synthesizer (CEM, UK). The standard 9-fluorenylmethoxycarbonyl (Fmoc) protection chemistry on a 4-methylbenzhydrylamine (MBHA). Rink amide resin (Novabiochem Corporation, UK) was employed. PAs were purified using preparative high-performance liquid chromatography (Waters, USA) with a reverse-phase Xbridge C18 column (Waters, USA) and a water/acetonitrile (0.1% NH_4_OH or TFA) binary mobile phase.

### Chick Chorioallantoic Membrane (CAM) Assay

#### Implantation, Extraction, and Chalkley Score

Animal studies were performed in accordance with the guidelines and regulations laid down in the Animals (Scientific Procedures) Act 1986. CAM model was carried out in accordance with Home Office Approval, UK (Project license—PPL P3E01C456). Chicken eggs were acquired from Medeggs (Norfolk, UK). Eggs were stored in a Hatchmaster incubator (Brinsea, UK) at 37 °C in a 60% humidified atmosphere and 1 h rotation. To ensure the maintenance of a humidified environment in the egg incubator, deionized water (DW) was supplemented every 2 days. Implantation was carried out after 7 days of incubation. To assess embryo viability and development, eggs were candled. A window of 1 cm^2^ was created with a scalpel onto the egg shell exposing the chorioallantoic membrane. Hydrogels were implanted, and the window was sealed with a sterile Parafilm strip (Bemis, Parafilm M, Laboratory Wrapping Film, Fisher Scientific, UK). Eggs were return to the Hatchmaster incubator for 7 days (37 °C in a 60% humidified atmosphere) without rotation. Chalkley scoring was used as previously described^3^ to quantify infiltration of blood vessels through the implanted scaffolds. Implants and blank controls were observed *in situ* under a stereo light microscope. A total of five independent counts obtained from the number of vessels fitting with the Chalkley graticule projected onto the samples were registered.

#### Histological Analysis

Integrated hydrogel samples were extracted and fixed in 4% paraformaldehyde (PFA) overnight. Samples were further embedded in optimum cutting temperature (OCT embedding matrix, CellPath, UK) and stored at −80 °C. Samples were sectioned using a Cryostat (CM 1850, Leica Biosystems, Germany), and 8 μm thick sections were collected using Kawamoto’s film method.^4^ Stainings (Goldner’s Trichrome and Von Kossa) were subsequently carried out on the cryotape. Sections were mounted using Super Cryomounting Medium (SCMM) type R3 (Section LAB, Co. Ltd. Japan) and UV cured for 30 min to photopolymerize the SCMM. Slides were imaged the following day using a Zeiss Axiovert 200 (Carl Zeiss, Germany).

## Supporting Information Available

Detailed explanation of the experimental methods and additional figures (PDF)
