# Supplementary material for: De Novo Design of Functional Coassembling Organic–Inorganic Hydrogels for Hierarchical Mineralization and Neovascularization
Source: ACS Nano. 2021 Jun 28;15(7):11202–17. doi: 10.1021/acsnano.0c09814 (PMC8320236; doi:10.1021/acsnano.0c09814)
Supplement: Supplementary file 1 — nn0c09814_si_001.pdf [file nn0c09814_si_001.pdf]

De Novo **Design of Functional Co-Assembling Organic-Inorganic Hydrogels for Hierarchical Mineralization and Neovascularization**

Babatunde O. Okesola[1,2], Ana Karen Mendoza-Martinez[1,2], Gianluca Cidonio[3,4], Burak Derkus[1,2,5], Delali K. Boccorh[6], David Osuna de la Peña[2], Sherif Elsharkawy[7], Yuanhao Wu[8,9], Jonathan I. Dawson[3], Alastair W. Wark[6], Dafna Knani[10], Dave J. Adams[11], Richard O. C. Oreffo[3], Alvaro Mata[1,2,8,9,12*]

1. Institute of Bioengineering, Queen Mary University of London, London, E1 4NS, UK.
2. School of Engineering and Materials Science, Queen Mary University of London, London, E1 4NS, UK.
3. Bone and Joint Research Group, Centre for Human Development, Stem Cells and Regeneration, Institute of Developmental Sciences, University of Southampton, Southampton, SO16 6YD, UK.
4. Center for Life Nano- and Neuro- Science (CL2NS), Fondazione Istituto Italiano di Tecnologia, 00161, Rome, Italy.
5. Department of Chemistry, Faculty of Science, Ankara University, 06560 Ankara, Turkey.
6. Department of Pure and Applied Chemistry, Technology and Innovation Centre, University of Strathclyde, Glasgow, G1 1RD, UK.
7. Centre for Oral, Clinical, and Translational Sciences, Faculty of Dentistry, Oral, and Craniofacial Sciences, King’s College London, London, SE1 1UL, UK.
8. School of Pharmacy, University of Nottingham, Nottingham, NG7 2RD, UK.
9. Biodiscovery Institute, University of Nottingham, Nottingham, NG7 2RD, UK.
10. Department of Biotechnology Engineering, ORT Braude College, Karmiel 2161002, Israel.
11. School of Chemistry, College of Science and Engineering, University of Glasgow, Glasgow, G12 8QQ, UK.
12. Department of Chemical and Environmental Engineering, University of Nottingham, Nottingham, NG7 2RD, UK.

Corresponding author: a.mata@nottingham.ac.uk

Supplementary Information Contents:

1. **Zeta potential and size distribution measurements**
2. **Circular dichroism spectroscopy**
3. **Small-Angle Neutron Scattering (SANS) analysis of hydrogel nanostructures**
4. **Atomic Force Microscopy (AFM)**
5. **Transmission Electron Microscopy (TEM) and High Resolution TEM (HRTEM)**
6. **Molecular dynamic simulations**
7. **Preparation of hydrogels**
8. **Dynamic rheological measurements**
9. **Characterization of surface properties of xerogels**
10. **Biomineralization of hydrogels**
11. **Monitoring of biomineralization process *via* Raman Spectroscopy**
12. **Synthesis and purification of peptide amphiphiles**
13. **Cytocompatibility and cell proliferation assessments**
14. **Chick chorioallantoic membrane (CAM) assay**
15. **References**

Materials and methods

1.0 Zeta potential (ζ)

All ζ-potential measurements were performed after resuspension of the PAs at a concentration of 1mM in ultrapure water. After loading the samples in folded capillary cells, measurements were performed at 25°C using a ζ-sizer instrument (Nano-ZS Zen 3600, Malvern Instruments, UK). For each PA, three separate samples were measured with at least five runs per sample.

2.0 Circular dichroism spectroscopy

Circular dichroism (CD) was measured with Chirascan™ circular dichroism spectrometer (Applied Photophysics Limited, UK) using quartz cell with 1 mm path length and the following parameters: data pitch – 0.5 nm, scanning mode – continuous, scanning speed – 100 nm/min, bandwith – 2 nm and accumulation - 5. All CD data are presented as ellipticity and recorded in millidegree (mdeg). CD measurements were performed on aqueous solutions of **PAH3** (0.01% w/v), **Lap** (0.025% w/v) and their mixtures. CD spectra were obtained by signal integrating 3 scans, from 190 to 260 nm at speed of 50 nm/min. Data were processed by a simple moving average and smoothing method.

3.0 Small-Angle Neutron Scattering (SANS) analysis of hydrogel nanostructures

The required concentration of **PAH3** (1% w/v), **Lap** (2.5% w/v) solutions and equal volume mixtures of **PAH3** (2% w/v) and **Lap** (5% w/v) were prepared in D_2O (400 µL). The solutions were transferred to 1 mm path length UV spectrophotometer grade quartz cuvettes (Hellma). The hydrogels of **PAH3_Lap** were prepared by mixing equal volume of **PAH3** (2% w/v) and **Lap** (5% w/v) in the cuvettes and gelation was allowed to proceed overnight. Synchrotron small-angle neutron scattering (SANS) measurements were performed on the fixed-geometry, time-of-flight LOQ diffractometer (ISIS Neutron and Muon Source, Oxfordshire, UK). A white beam of radiation with neutron wavelengths spanning 2.2 to 10 Å was enabled access to Q [$Q = 4\pi\sin(\theta/2)/\lambda$] range of 0.004 to 0.4 $Å^{-1}$ with a fixed-sample detector distance of 4.1 m. The cuvettes were mounted in aluminium holders. Time taken for each measurement was approximately 30 minutes. All scattering data were normalized for the sample transmission, the backgrounds were corrected using a quartz cell filled with D_2O and the linearity and efficiency of the detector response was corrected using the instrument-specific software.

For all of the samples, the data at very low Q was ignored for the fitting. A Q-range of $0.005<Q<0.5$ Å^{-1} was used to fit the data. The background level varied between samples. For the fittings, the background was generally fixed and not varied as part of the fit. The data for the Lap alone possesses a $Q^{-1.8}$ dependency in the range $0.01<Q<0.1$ Å^{-1}, which indicates a disk-shaped object (where one would expect a Q^{-2} dependency) (see graph Supplementary Figure S2). The data are similar to those reported elsewhere.[1] As discussed in this paper, there is also the start of a Q-independent plateau at approximately Q=0.012 Å^{-1}, implying that the largest particle dimension is approximately 500 Å. The data were fitted to a cylinder model, with the fit in agreement with thin disks with a thickness of around 12 Å. A SLD of 4.18×10^{-6} Å^{-2} was used on the basis of previous data. Data for **PAH3** were fitted to a cylinder model, although it is necessary to include some polydispersity in radius to satisfactorily fit the data. A SLD of 1.51×10^{-6} Å^{-2} was used (from the NIST calculator). For **PAH3** combined with Lap, the data can be fitted to a combination of two cylinders (one long and one flat (or disk) cylinder). Details of the parameters for all the fittings are presented in **Table S1**.

4.0 Atomic Force Microscopy (AFM)

AFM was performed on the Bruker Multimode 8 AFM with a Nanoscope V controller using PeakForce Tapping mode with a ScanAsyst Air cantilever (spring constant 0.4N/m). The cantilever was calibrated using the automated 'no touch' calibration routine built into the software. Solutions of **PAH3** (0.01% w/v, 40 µL), Lap (0.025% w/v, 40 µL) and **PAH3**/**Lap** mixtures were dropped onto freshly cleaved mica surface. The samples were air dried at room temperature for 24 h and imaged with a PeakForce setpoint of 500pN with a PeakForce amplitude of 30nm and frequency of 4kHz. Images were acquired at 512x512 pixels at a line rate of 2.8Hz. The height images were processed in the Nanoscope Analysis software after using 1st order flattening to remove tilt. Images were processed in Nanoscope 1.7.

5.0 Transmission Electron Microscopy (TEM) and High Resolution TEM (HRTEM)

Aqueous solutions of **PAH3** (0.01% w/v) and Lap (0.025% w/v, exfoliated with 0.0068% w/v ASAP) were dissolved ultrapure water. Similarly, mixtures of **PAH3** (0.02% w/v) and **Lap** (0.05% w/v) were also prepared. Samples were mounted on copper TEM plasma etched holey carbon-coated copper grid (Agar Scientific, Stansted, UK). The grids were immersed in the sample solutions for five minutes. Excess was removed on filter paper before incubation with 2% uranylacetate solution for 30 seconds. Grids were then washed with ultrapure water for 30s

and air dried for 24h at room temperature. Bright-field TEM imaging was performed on a JEOL 1230 Transmission Electron Microscope operated at an acceleration voltage of 80 kV. All the images were recorded by a Morada CCD camera (Image Systems). At least three images were taken per samples for further analysis.

High resolution transmission electron microscope (HRTEM) images, selected area electron diffraction (SAED) patterns, scanning transmission electron microscope (STEM) images and energy dispersive X-ray spectroscopy (EDS) spectrum images were obtained with an FEI Talos F200X microscope equipped with an X-FEG electron source and Super-X SDD EDS detectors. The experiment was performed using an acceleration voltage of 200kV and a beam current of approximately 1 nA. TEM Images were recorded with a FEI CETA 4k x 4k CMOS camera. STEM images were acquired with HAADF and BF detectors.

6.0 Molecular dynamic simulations

The dynamic simulation was conducted according to the following steps:

Step 1*: Building layered cells*

Layered sepiolite

A simulation box containing 2 layers of sepiolite was constructed using the Build Layer tool. The structure was built as a crystal with a vacuum region of 20Å between the layers. A lattice with dimensions of 13.4x26.8x37.64 Å was obtained. The motion of the atoms of the lattice was constrained using the Edit Constraints dialog and choosing Fix Cartesian position. Thus, the XYZ position property in Cartesian space was held constant for the lattice atoms during the dynamic simulations, and only the PAs molecules, which were inserted later, were able to move.

Supercell layered sepiolite

To increase the surface area of the sepiolite crystal a supercell was built. A supercell has lattice vectors which are integral multiples of their equivalents in the original lattice, with a P1 (crystal) symmetry. The supercell was constructed from the sepiolite crystal using the Symmetry tool in the Built function with a factor of 2 in the (a) and (b) directions. Afterwards, a layered supercell sepiolite was constructed according to the procedure described above (at Layered sepiolite). A lattice with dimensions of 26.8x53.6x37.64 Å was obtained.

Insertion of PAs molecules into the layered sepiolite cells

Each of the three PAs examined (**PAH3**, PAK3 and PAE3) were inserted into the layered cell:

- One molecule of each into the small Layered sepiolite cell

- Four molecules of each into the Layered sepiolite supercell
- Ten molecules of each into the Layered sepiolite supercell

After the insertion, the cells were optimized using the Geometry Optimization tool of Forcite module with COMPASII forcefield.

Step 2: *Molecular Dynamics simulation*

Dynamic simulation was performed at 298 K. The cells were subjected to 10^6 dynamic steps of 1fs each at constant moles number, volume and temperature (NVT ensemble). All MD simulations were conducted using Forcite module with COMPASSII force field. Electrostatic and van der Waals terms were considered using Atom based summation methods with a repulsive cut-off of 12.5 Å.

Step 3: *Analysis*

The resulted dynamic trajectories (1000 frames) were analyzed using Forcite module analysis tools.

Interaction energy

The interaction energy between the clay and PAs can be calculated according to the equation:

$$\mathbf{E_{Interaction} = E_{total} - (E_{clay} + E_{PA})}$$

E_{total} is the energy of the clay and the PA, E_{clay} is the energy of the clay without the PA and E_{PA} is the energy of the PA without the clay. These calculations are all single point energies and they were undertaken whilst constraints are removed. A negative value indicates that the PA is binding to the clay.

7.0 Preparation of hydrogels

Aqueous solution of **Lap** (2.5% w/v) was prepared by adding the requisite amount of Laponite powder to a stirred suspension of ASAP (0.06% w/v) in ultrapure water. The **Lap** suspension was sonicated for 30 min until clear transparent sample was obtained. Aqueous solutions of PA (2% w/v) were prepared in HEPES buffer. **PA-Lap** hydrogels were prepared by injecting solution of PA (20 µL) into a larger volume of Lap (100 µl). Gelation was allowed to proceed overnight at room temperature. Hydrogels of **PAH3** (2% w/v) were prepared by basifying aqueous solution of **PAH3** with NaOH (1M).

8.0 Dynamic rheological measurements

Rheological measurements were performed using a Discovery Hybrid Rheometer, Rheo-DHR3 (TA Instruments). All data were collected at 25 °C. The preformed hydrogels were added to the center of the bottom plate and the top parallel plate (with 8 mm diameter) was lowered to a gap of 100 µm. The amplitude sweep measurements were performed between 0.1 and 50 % strain at constant frequency (1 Hz). Similarly, frequency sweep rheographs were obtained between 0.1 and 20 Hz at constant strain (0.5%). Self-healing was assessed initially at 0.1% strain for 100 s, then at 100% strain for 200 s, 0.1% strain for 200 s, 100% strain for 200 s and 0.1% strain for 400 s.

9.0 Characterization of surface properties of xerogels

Nitrogen sorption isotherms of the lyophilized xerogels were measured at 77 K using an Autosorb-IQ system (Quantachrome Instrument, USA). Before measurements, the samples were degassed in a vacuum at 120 °C overnight. The specific surface areas (S_{BET}) were calculated by the multipoint Brunauer-Emmet-Teller method using adsorption data in a relative pressure range from 0.04 to 0.2, and the pore-size distribution was calculated based based on quenched solid density function theory (QSDFT) using the adsorption branches of isotherms assuming slit and cylindrical pores geometries. By using the Barrett-Joyner-Halenda (BJH) model, the mesoporous surface areas (S_{BJH}) were calculated from the adsorption line. The microporous surface areas (S_{DR}) were calculated from the adsorption line by the Dubinin–Radushkevich (DR) model.

10.0 Biomineralization of hydrogels

The mineralizing solutions were prepared as previously reported by Elsharkawy *et al*.[2] Briefly, an aqueous suspension of hydroxyapatite powder (2 mM) and sodium fluoride (2 mM) was prepared in deionized water with continuous stirring. Then, 69% nitric acid was added dropwise to the suspension to aid a complete dissolution of the hydroxyapatite precipitates at pH 2.4. Thereafter, an aqueous solution of ammonium hydroxide (30%) was added dropwise to the hydroxyapatite solution until it reaches pH 6. Various hydrogels were then immersed in the hydroxyapatite solutions and incubated for eight days at 37 °C using a temperature-controlled incubator (LTE Scientific, Oldham, UK).

11.0 Monitoring of biomineralization process via Raman Spectroscopy

All Raman analysis was carried out on a confocal WITEC Alpha300 system utilising a 785 nm laser and a 20× (S Plan Fluor, NA 0.45, ELWD) objective lens. Raman scatter was collected in a backscattering geometry. A small amount of each sample was placed on a microscope glass slide which had been previously cleaned with a methanol-soaked tissue, with a new slide used for each sample. The incident laser power was constant for all samples at 63 mW. No signal loss was observed, for example due to photobleaching or carbonisation, when samples were irradiated on the same spot in triplicate with integration times ranging from 10 s to 60 s. All spectra processing was performed using SpectraGryph 1.2 involving: 1. cosmic ray removal, 2. background correction and then 3. subsequent normalization. An advanced baseline correction protocol available in the SpectroGryph software was applied which fits a polynomial curve to the spectral regions where there is no Raman peak and enables subtraction of the variable y-offset associated with the luminescence background. To enable comparison of the relative changes in the Raman intensity of the 1047 cm^{-1} peak in Figures 6 and S10, all spectra were normalized with respect to the peak intensity in the 2800-3000 cm^{-1} region. This approach was adopted as the integration time was varied between samples to optimize the signal to noise alongside variation in background luminescence with mineralization times. However, the C-H vibrational spectral shape across 2800-3000 cm^{-1} remained relatively unchanged for each sample and the Raman peak intensity was also observed to change proportionally with integration time in this region. For each measurement, multiple spectra were acquired across the sample with the focus depth also optimized, which revealed good uniformity and ensured that the spectra presented are representative of the sample.

12.0 Synthesis and purification of peptide amphiphiles

The peptide amphiphiles (PAs) were synthesized using solid phase peptide synthesis (SPPS) on Liberty Blue automated microwave peptide synthesizer (CEM, UK). The standard 9-fluorenylmethoxycarbonyl (Fmoc) protection chemistry on a 4-methylbenzhydrylamine (MBHA). Rink Amide resin (Novabiochem Corporation, UK) was employed. Amino acid couplings were performed using 4 mmol equivalent of Fmoc-protected amino acids (Novabiochem Corporation, UK), 4 mmol equivalents of 1-hydroxybenzotriazol (HOBT, Carbosynth Limited, UK) and 6 mmol equivalents of *N,N'*-diisopropylcarbodiimide (DIC, Sigma–Aldrich, UK) for 1h. Fmoc deprotections were performed with 20% piperidine (Sigma-Aldrich, UK) in *N,N*-dimethylformamide (DMF, Alfa Aesar, UK). Following Fmoc removal from the final amino acid residue, the alkyl tail moiety (from palmitic acid, $C_{16}H_{32}O_2$, Calbiochem, UK) was conjugated to the free N-terminus. The alkylation reaction was

accomplished by using palmitic acid (4 mmol), HOBT (4 mmol), and DIC (6 mmol) in DMF/dichloromethane. The reaction was allowed to proceed at room temperature for 4 h or until obtaining a negative Kaiser test. Cleavage of the PA cleavage from the resin and deprotection of the side chains was done with a mixture of trifluoracetic acid (TFA, Sigma-Aldrich, UK), triisopropylsilane (TIS, Alfa Aesar, UK) and water in a ratio of 95:2.5:2.5 for 3h at room temperature. After filtration of the cleavage mixture and two subsequent TFA washings, TFA was removed by rotary-evaporation and the resulting solution was triturated with cold diethylether. The precipitate was collected by centrifugation, washed twice with cold diethylether, air-dried, dissolve in deionised water and lyophilized. The crude PA powder (100 mg) was dissolved in water (10 ml) with the addition of NH_4OH (0.1%) or TFA (0.1%) and was then purified using a preparative High-Performance Liquid Chromatography (Waters, USA) with reverse-phase Xbridge C18 column (Waters, USA) and water/acetonitrile (0.1% NH_4OH or TFA) binary mobile phase. The HPLC fractions were rotavap to remove the acetonitrile. Finally, the PAs were lyophilized to obtain a white fluffy pure powder. PAs were characterized by electrospray ionization mass spectrometry (ESI-MS). Yield: 80%

13.0 Cytocompatibility and cell proliferation assessment

A LIVE-DEAD® Cytotoxicity Assay Kit (Invitrogen, USA) was used to assess the cytocompatility human bone marrow derived mesenchymal stem cells (P2, generous supply from Prof Oreffo's lab) seeded on various hydrogels and tissue culture plastics (TCP as a control) at $1x10^6$ cells/mL. Cells were cultured on the hydrogels and TCP in growth media consisting of Dulbecco's Modified Eagle's Medium (DMEM) supplemented with 10% Fetal Bovin Serum (FBS), 1% L-glutamine and 1% antibiotic–antimycotic solution. The cultured cells were incubated under standard conditions (5% CO_2, 95% humidity and 37 °C). Cytotoxicity was assessed at various time intervals day 1, 3 and 7 using calcein AM (1 μM) and ethidium homodimer (2 μM) in PBS. Fluorescence images were captured on laser scanning confocal and multiphoton microscopy (TCS SP2, Leica Microsystems, Germany). Live cells were stained green with calcein AM and dead cells red with ethidium homodimer.

Proliferation of cells on various hydrogels and TCP was assessed by colorimetric method using 3-(4,5-dimethylthiazol-2-yl)-5-(3-carboxymethoxyphenyl)-2-(4-sulfophenyl)-2H-tetrazolium, inner salt; (MTS) (Promega). At various time points, the cell growth media was replaced with an aqueous solution of MTS following the manufacturer's protocol and incubated with the cells for 30 min. The absorbance was measured at 490 nm using a microplate reader (FLUOstar

Optima FL).

14.0 Chick chorioallantoic membrane (CAM) assay

Implantation, extraction and Chalkley score

Animal studies were performed in accordance with the guidelines and regulations laid down in the Animals (Scientific Procedures) Act 1986. CAM model was carried out in accordance with Home Office Approval, UK (Project license – PPL P3E01C456). Chicken eggs were acquired from Medeggs (Norfolk, UK). Eggs were stored in Hatchmaster incubator (Brinsea, UK) at 37 °C in a 60 % humidified atmosphere and 1 h rotation. To ensure the maintenance of a humidified environment in the egg incubator deionised water (DW) was supplemented every two days. Implantation was carried out after 7 days of incubation. To assess embryo viability and development eggs were candled. A window of $1cm^2$ was created with a scalpel onto the egg shell exposing the chorioallantoic membrane. Hydrogels were implanted and the window sealed with a sterile Parafilm strip (Bemis™, Parafilm M™, Laboratory Wrapping Film, Fisher Scientific, UK). Eggs were return to the Hatchmaster incubator for 7 days (37 °C in a 60 % humidified atmosphere) without rotation. Chalkley scoring was used as previously described[3] to quantify infiltration of blood vessels through the implanted scaffolds. Implants and blank controls were observed *in situ* under a stereo light microscope. A total of five independent counts obtained from the number of vessels fitting with the Chalkley graticule projected onto the samples were registered.

Histological analysis

Integrated hydrogel samples were extracted and fixed in 4 % paraformaldehyde (PFA) overnight. Samples were further embedded in optimum cutting temperature (OCT embedding matrix, CellPath, UK) and stored at -80 °C. Samples were sectioned using a Cryostat (CM 1850, Leica Biosystems, Germany) and 8µm thick sections were collected using Kawamoto's film method.[4] Stainings (Goldner's Trichrome and Von Kossa) were subsequently carried out on the cryotape. Sections were mounted using Super Cryomounting Medium (SCMM) type R3 (Section LAB, Co. Ltd. Japan) and UV cured for 30 min to photo-polymerise the SCMM. Slides were imaged the following day using a Zeiss Axiovert 200 (Carl Zeiss, Germany).

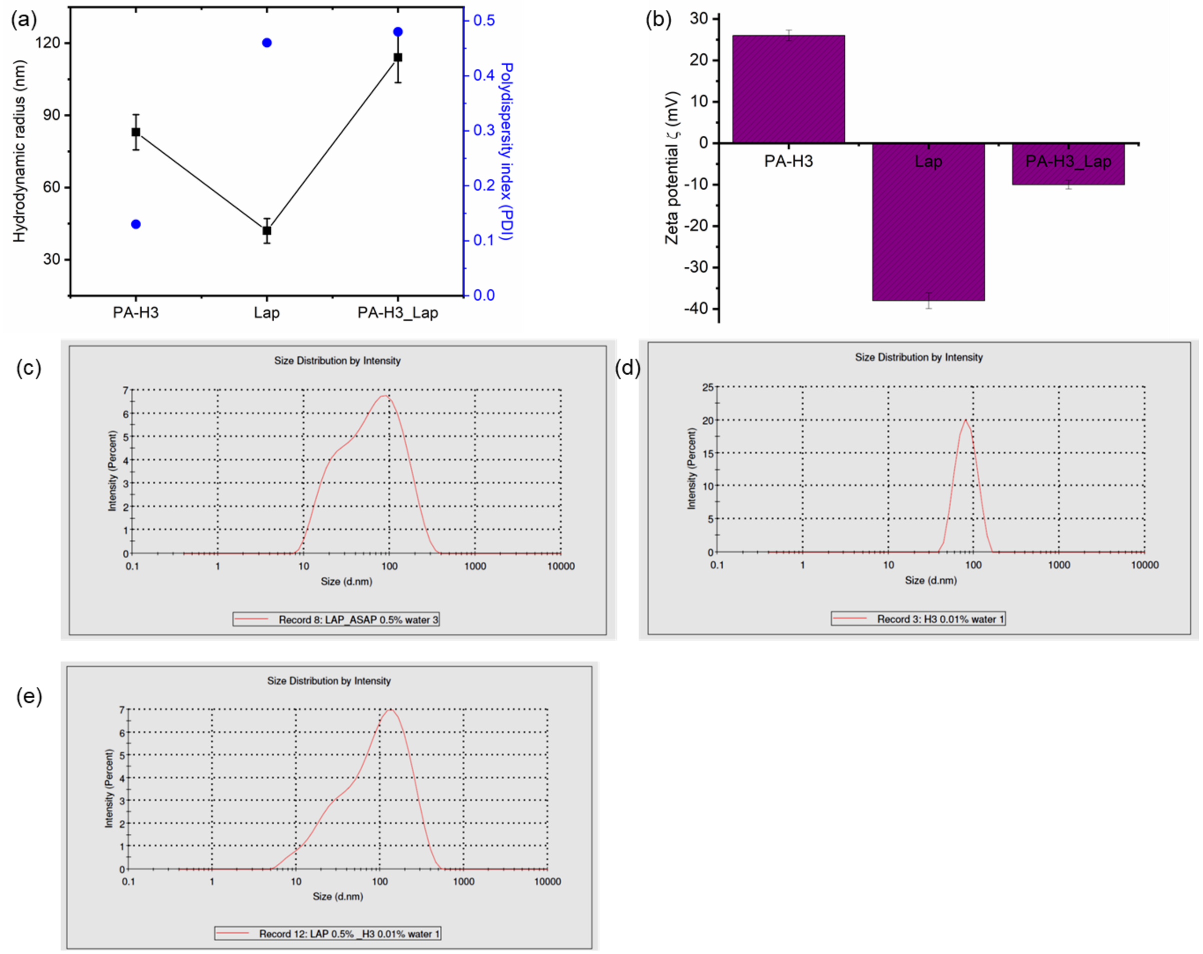

Supplementary Figure S1. (a) Hydrodynamic radius and polydispersity index for **PAH3**, exfoliated Lap and co-assembled **PAH3-Lap**. (**b**) Zeta potential values for aqueous solution of **PAH3**, exfoliated Lap and co-assembled **PAH3-Lap**. (c,d,e) size distribution of **PAH3** nanofibers, **Lap** nanodisks and **PAH3-Lap** nanofiber-nanodisk composites.

Supplementary Table S 1. Table showing fitting parameters for SANS data

	**Lap**	**PAH3**	**PAH3-Lap**
Background / cm^{-1}	0.004	0.001	0.003
Scale	$2.86x10^{-3} \pm 5.86x10^{-4}$		$3.95x10^{-3} \pm 2.89x10^{-4}$
Radius / Å	128.0 ± 1.9		134.7 ± 1.6
Length / Å	12.1 ± 2.5		12.0 ± 1.4
Scale		$1.457x10^{-3} \pm 4.214x10^{-6}$	$2.29x10^{-4} \pm 9.20x10^{-6}$
Radius / Å		37.9 ± 0.1	42.8 ± 0.4
Length / Å		3487 ± 121	7400 ± 400
Polydispersity		0.2	
χ^2	2.03	3.6982	2.3281

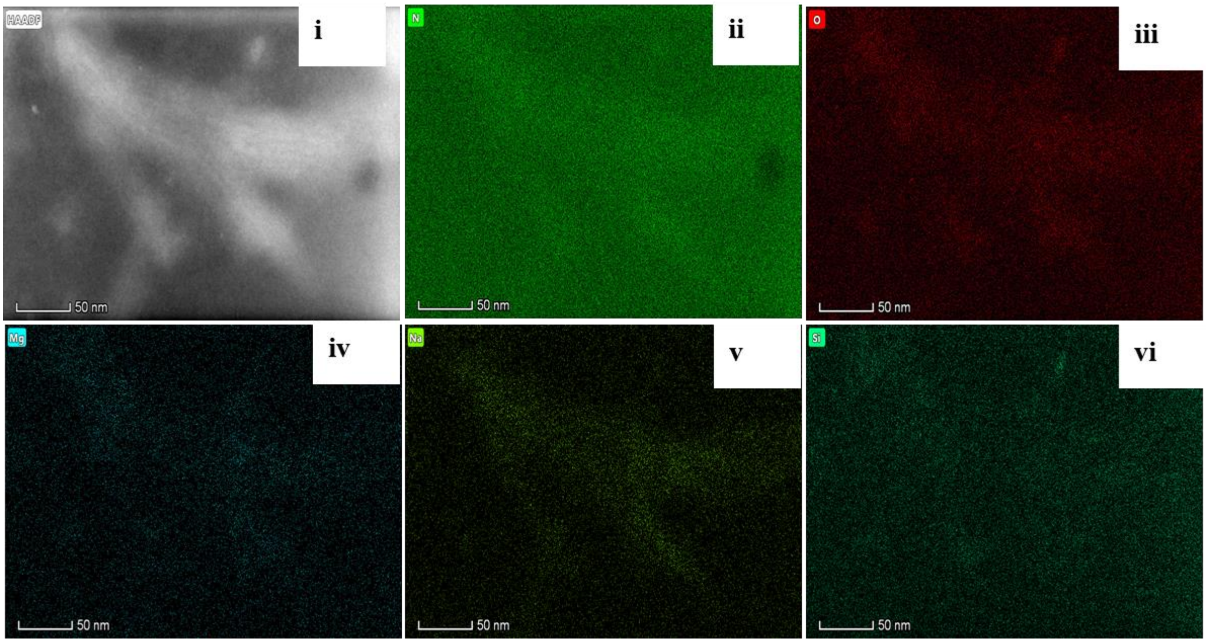

Supplementary Figure S2. HRTEM-EDS mapping showing bright field image of **PAH3-Lap** nanofiber-nanodisk co-assembly, which is evident from co-localization of nitrogen N (mainly from **PAH3**) and oxygen (O), magnesium (Mg), sodium (Na) and silicon (Si) from **Lap**.

Supplementary Table S 2. Table showing interaction energies of Sepiolite with **PAH3**, **PAK3**

and **PAE3**

	Interaction energy (kcal/mol)		
	Small cell<br>1 PA molecule	Supercell<br>4 PA molecules	Supercell<br>10 PA molecules
Sepiolite + **PAH3**	-736.38	-3060.72	-5626.32
Sepiolite + PAK3	-835.85	-2974.20	-3210.89
Sepiolite + PAE3	20668.67	82452.29	87478.13

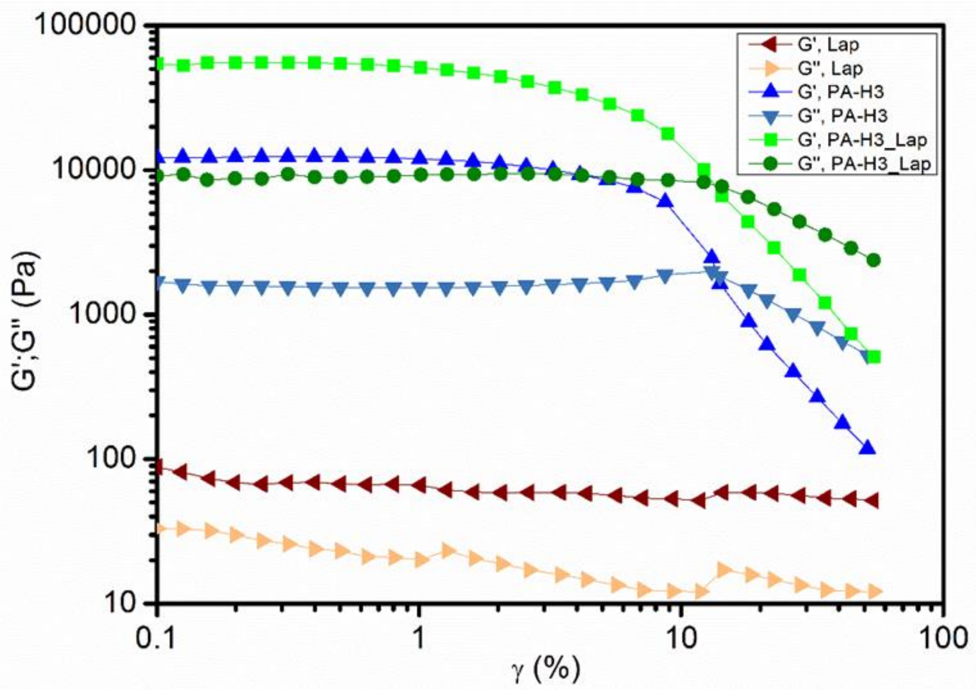

Supplementary Figure S3. Amplitude sweep rheographs showing strain-to-failure values for **Lap**, **PAH3** and **PAH3-Lap** hydrogels.

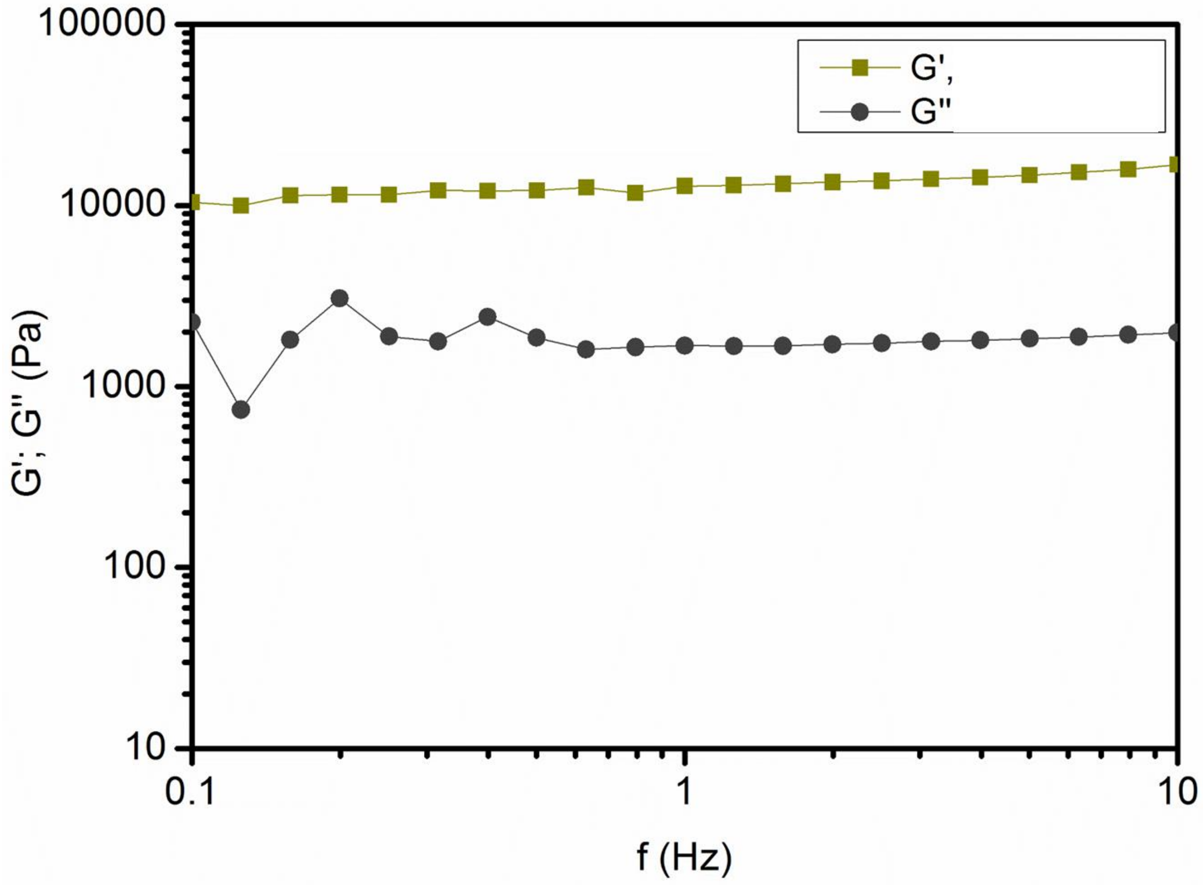

Supplementary Figure S4. Frequency sweep rheographs for **PAK3-Lap**.

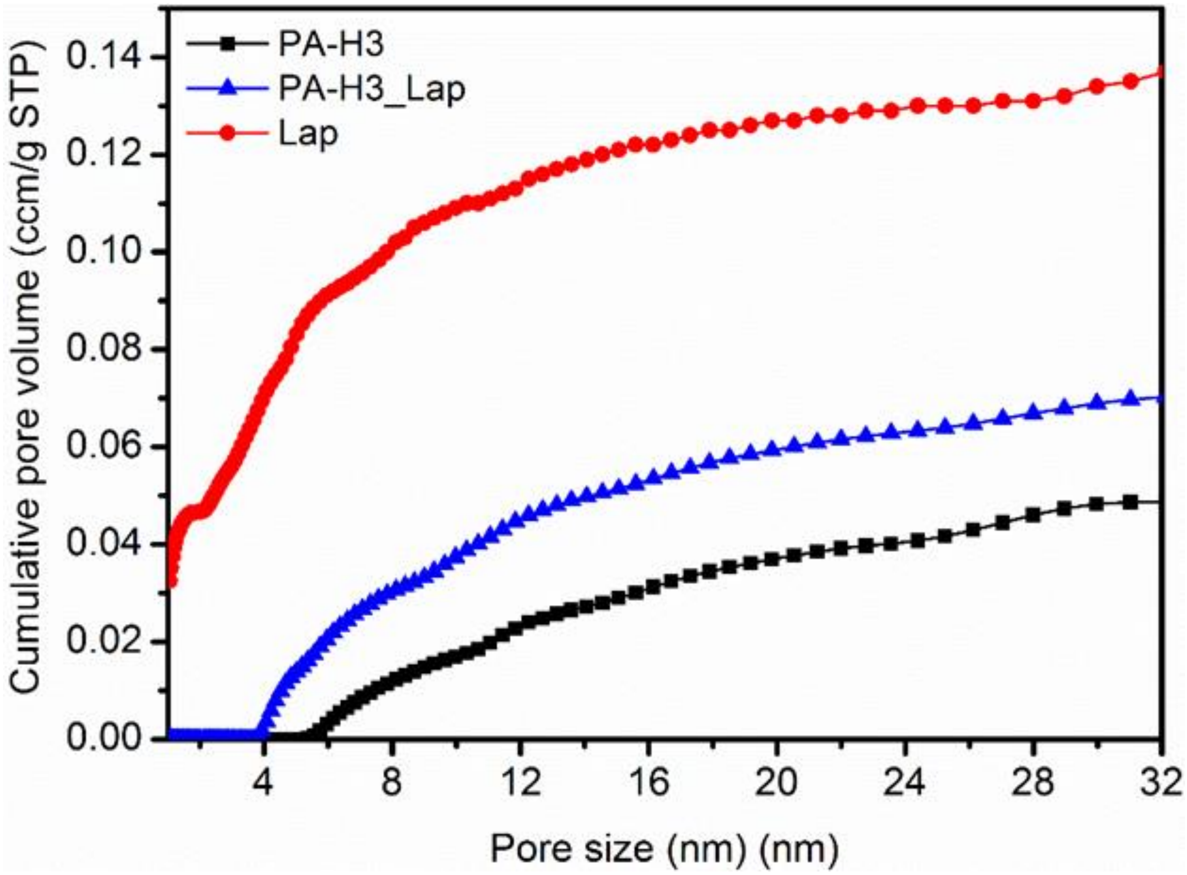

Supplementary Figure S5. Cumulated pore size distribution in xerogels of **Lap**, **PAH3**, **PAH3-Lap.**

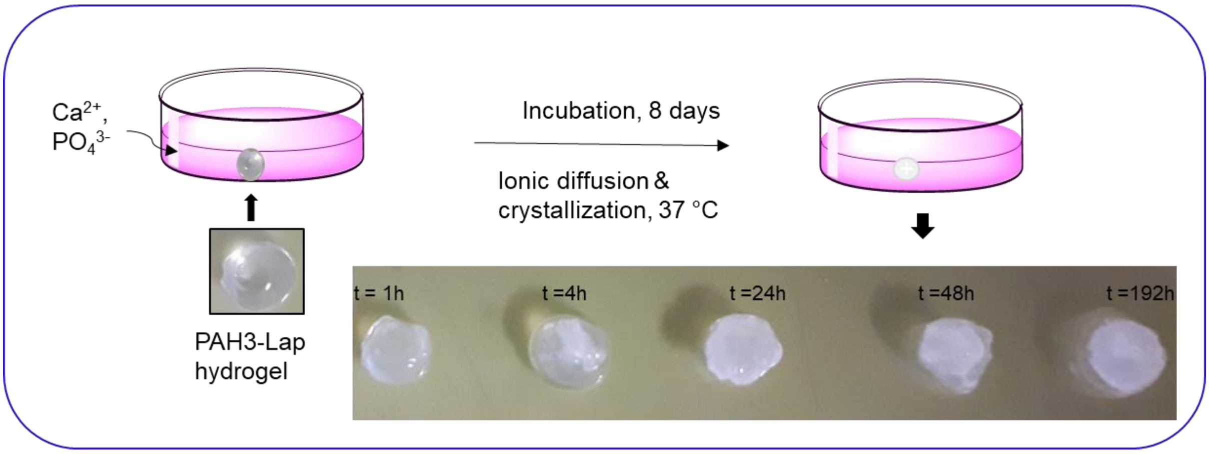

Supplementary Figure S6. Schematic representation of the procedure to mineralize **PAH3-Lap** hydrogels.

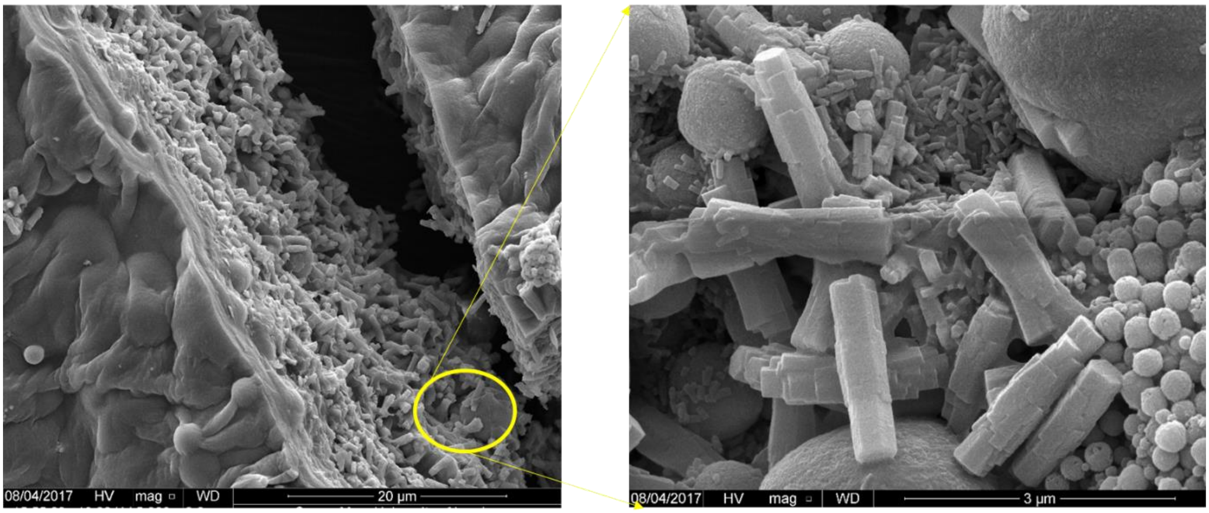

Supplementary Figure S7. Scanning electron micrographs of mineralized PAK3 after 8 days incubation in mineralizing solution

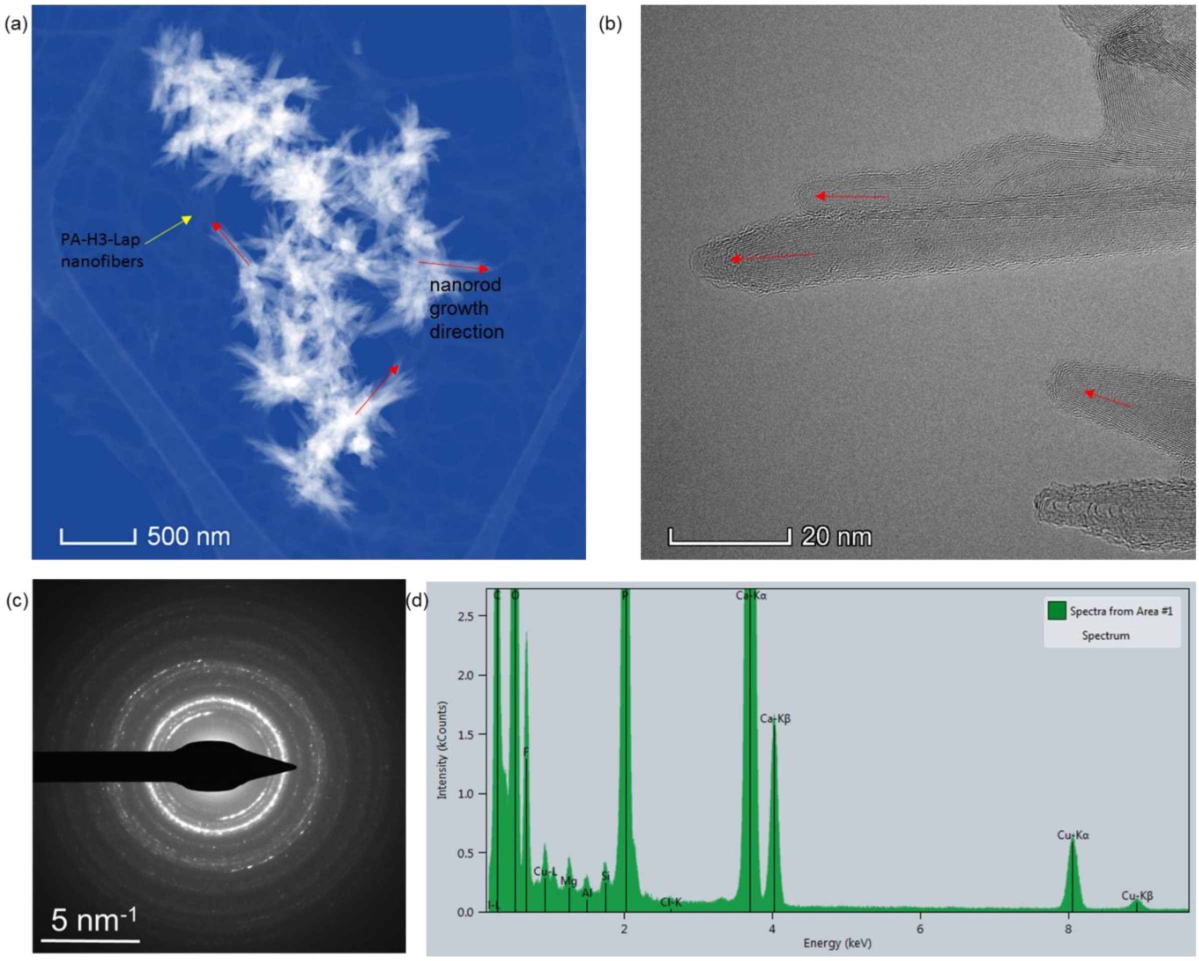

Supplementary Figure S8. (a) HRTEM bright field image after 2 h of mineralization showing the spherulitic growth of apaptite crystals (red arrow) within and along the nanofiber networks (yellow arrow) of **PAH3-Lap** hydrogels. (b) Enlarged selected area of HRTEM image of the apatite crystals showing an oriented crystal growth pattern. (c) FFT image of apatite crystals growing in **PAH3-Lap** hydrogels within 2h of incubation. (d) EDS spectrum for the mineralized **PAH3-Lap** hydrogels.

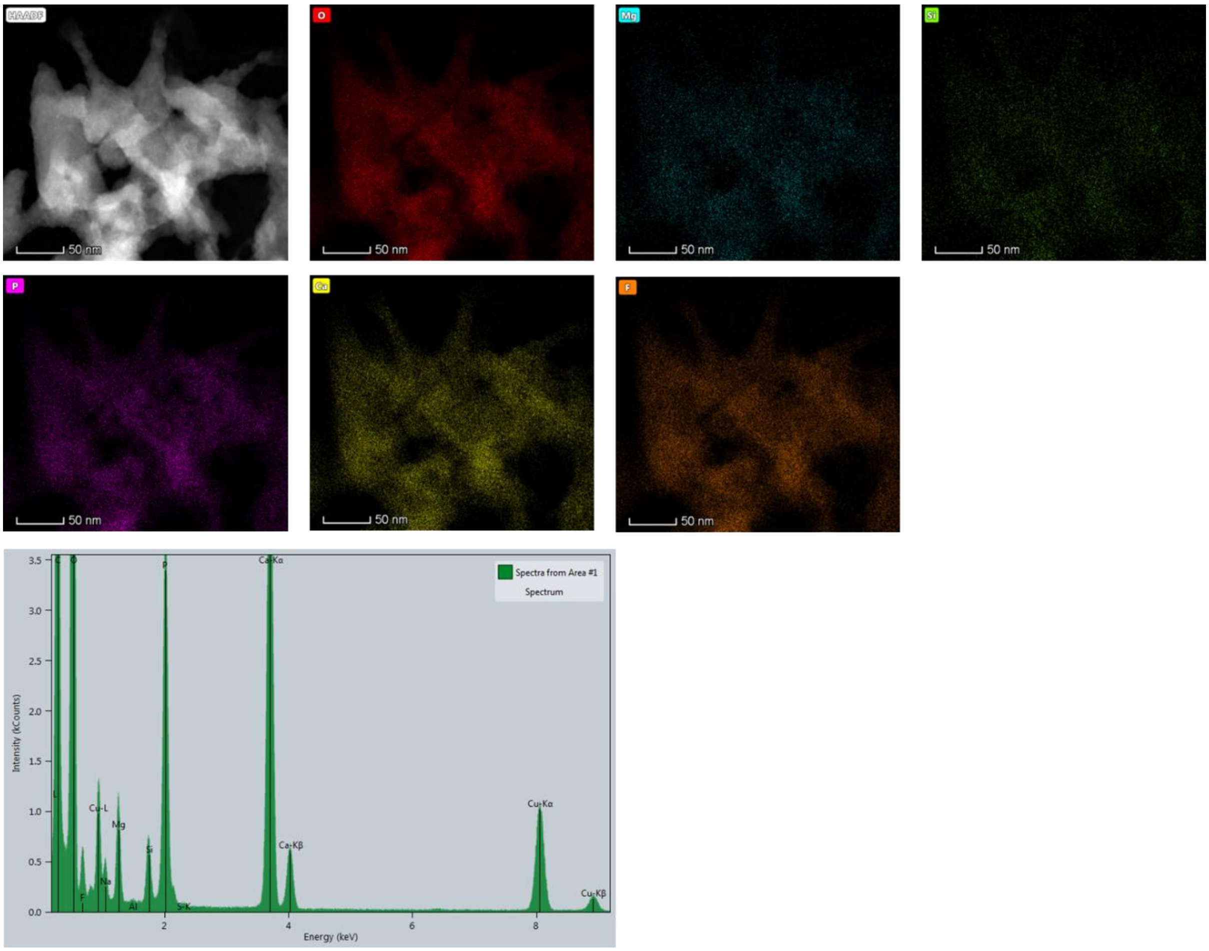

Supplementary Figure S9. (a) HRTEM-EDS micrographs showing a bright field image as well as elemental mapping of mineralized Lap suspension without **PAH3** after 8 days. (b) EDS spectra for the mineralized **Lap** suspension.

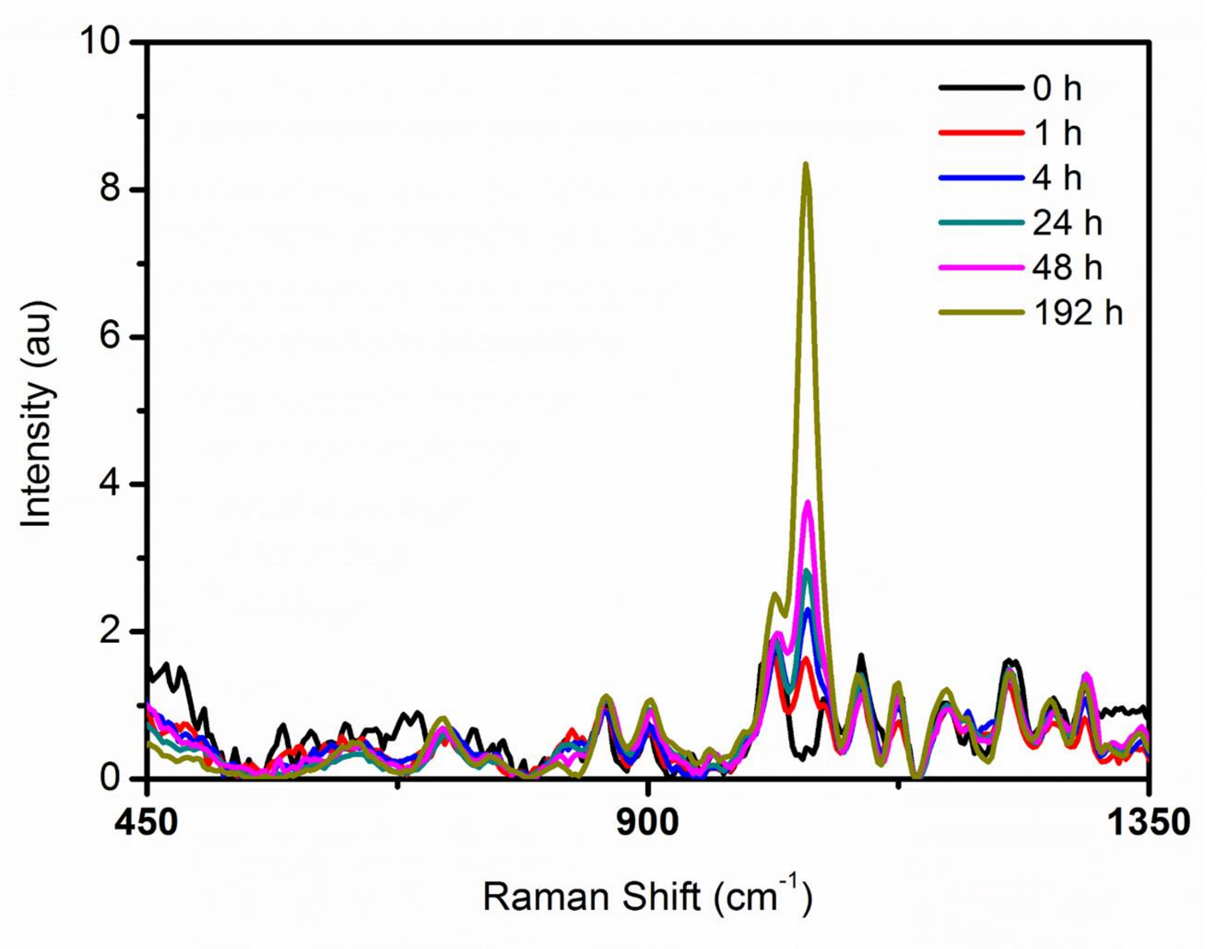

Supplementary Figure S10. Raman spectra showing time-resolved kinetics of crystal growth in **PAH3-Lap** hydrogels. Each spectrum has been background corrected and normalized with respect to the signal intensity at 2800-3000 cm^{-1} to aid comparison.

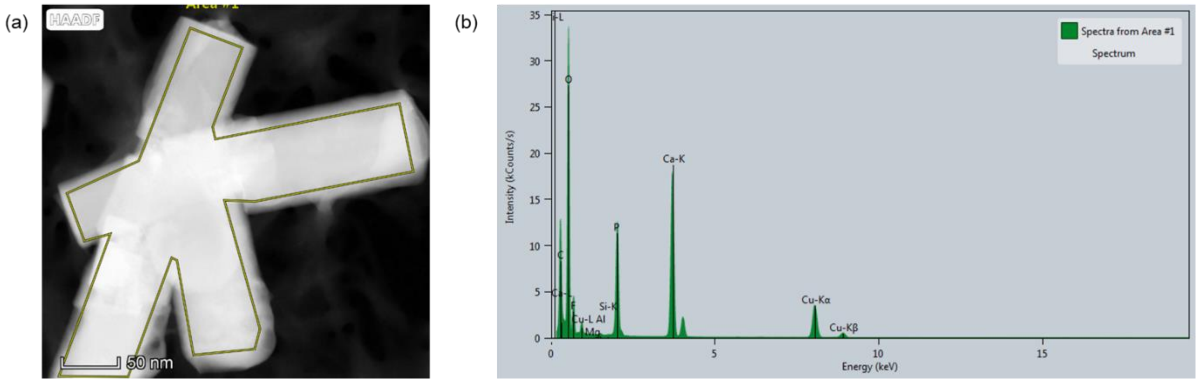

Supplementary Figure S11. (a) HRTEM bright field image of hydroxyapatite nanorod formed in **PAH3**-Lap hydrogels after 8 days and (b) EDS spectrum showing that the Ca/P ratio in the crystal was 1.69, which indicates the formation of hydroxyapatite crystals.

Supplementary Table S3. Identification and details of diffraction spots on the fast Fourier transform patterns (as shown in Fig. 6d_iii of the main paper) for the crystals formed in **PAH3-Lap** hydrogels.

$R=\frac{1}{2}D$ (1/nm)	1.235	3.412	2.907
$d=\frac{1}{R}\times 10$ (Å)	8.091	2.9325	3.437
Ref (HAP-PDF standard card)	8.115	3.067	3.437
(hkl)	(100)	(210)	(002)

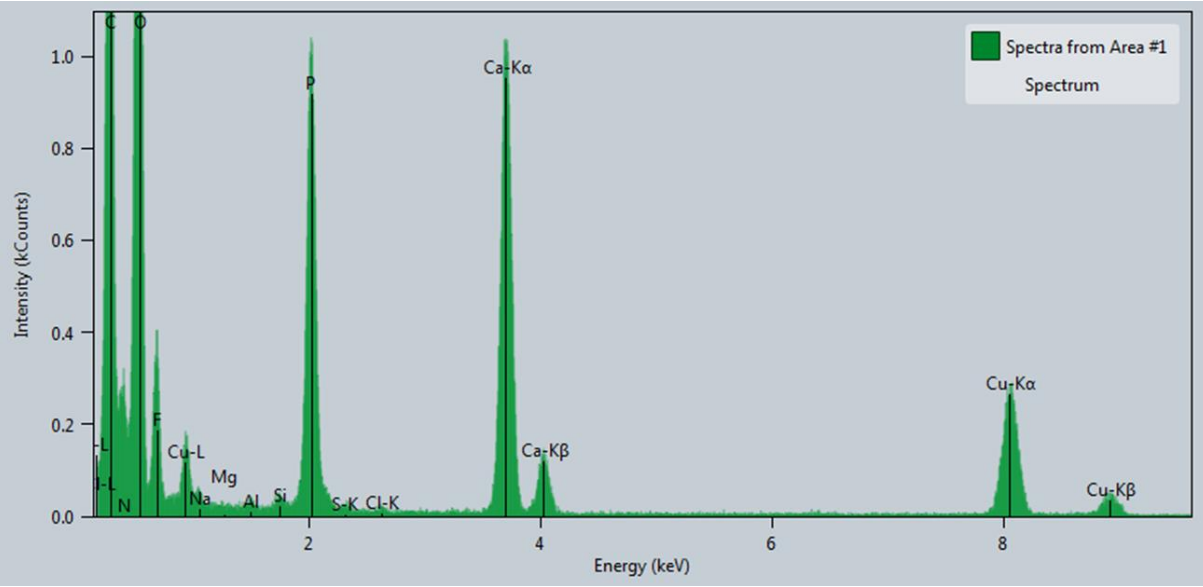

Supplementary Figure S12. EDS spectrum showing that the Ca/P ratio in the crystal formed in PAH3 hydrogels after 8 days incubation.

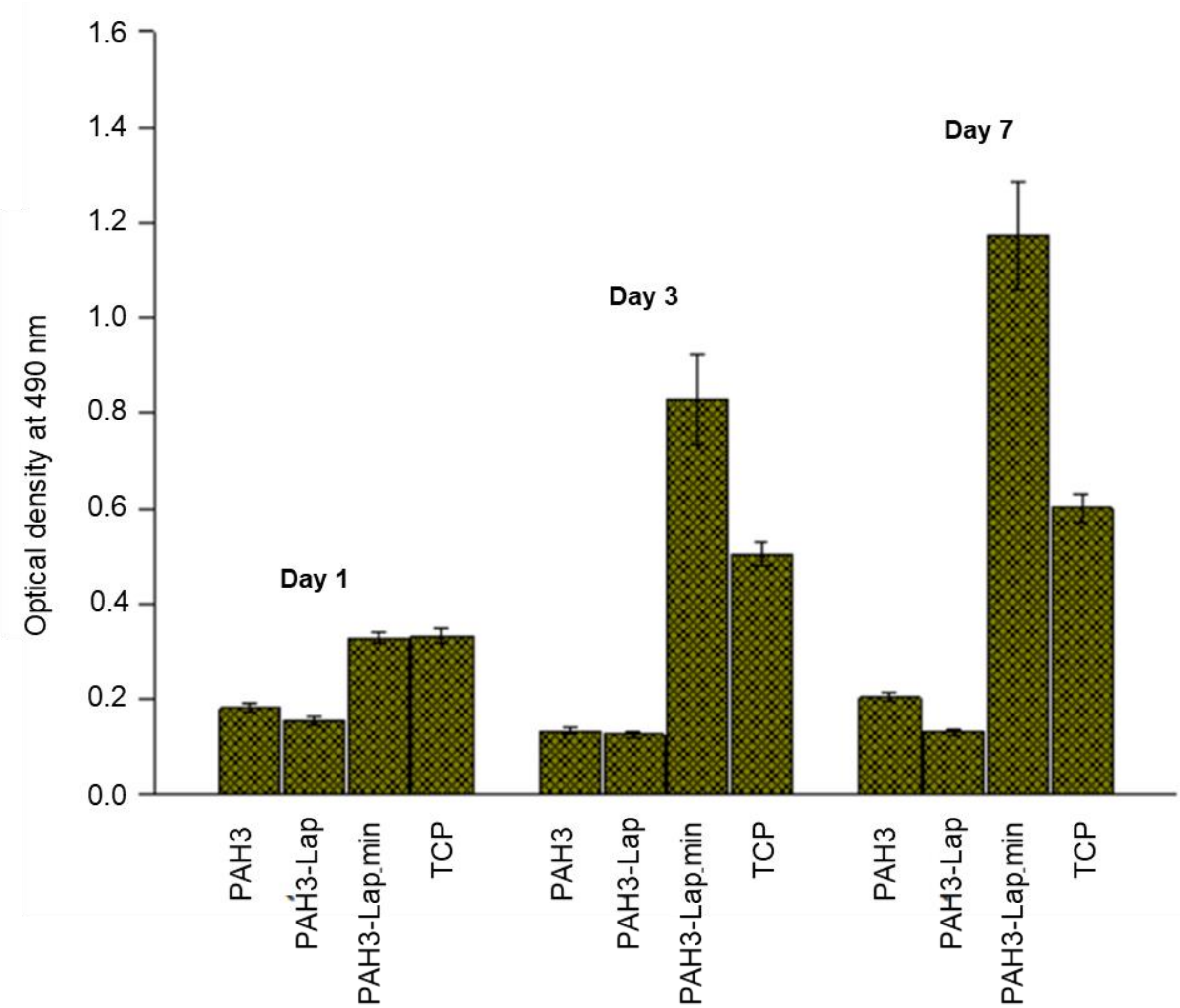

Supplementary Figure S13. Proliferation hBMSCs on hydrogels.

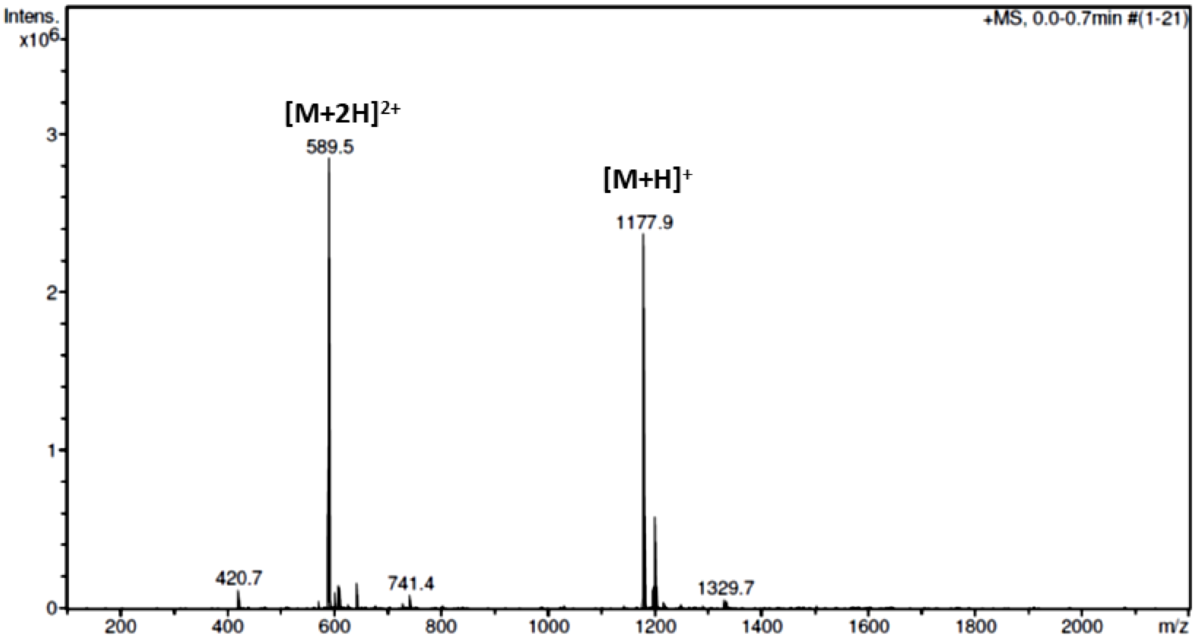

Supplementary Figure S14. ESI-MS of **PAH3** ions $[M+2H]^{2+}$ and $[M+H]^{+}$.

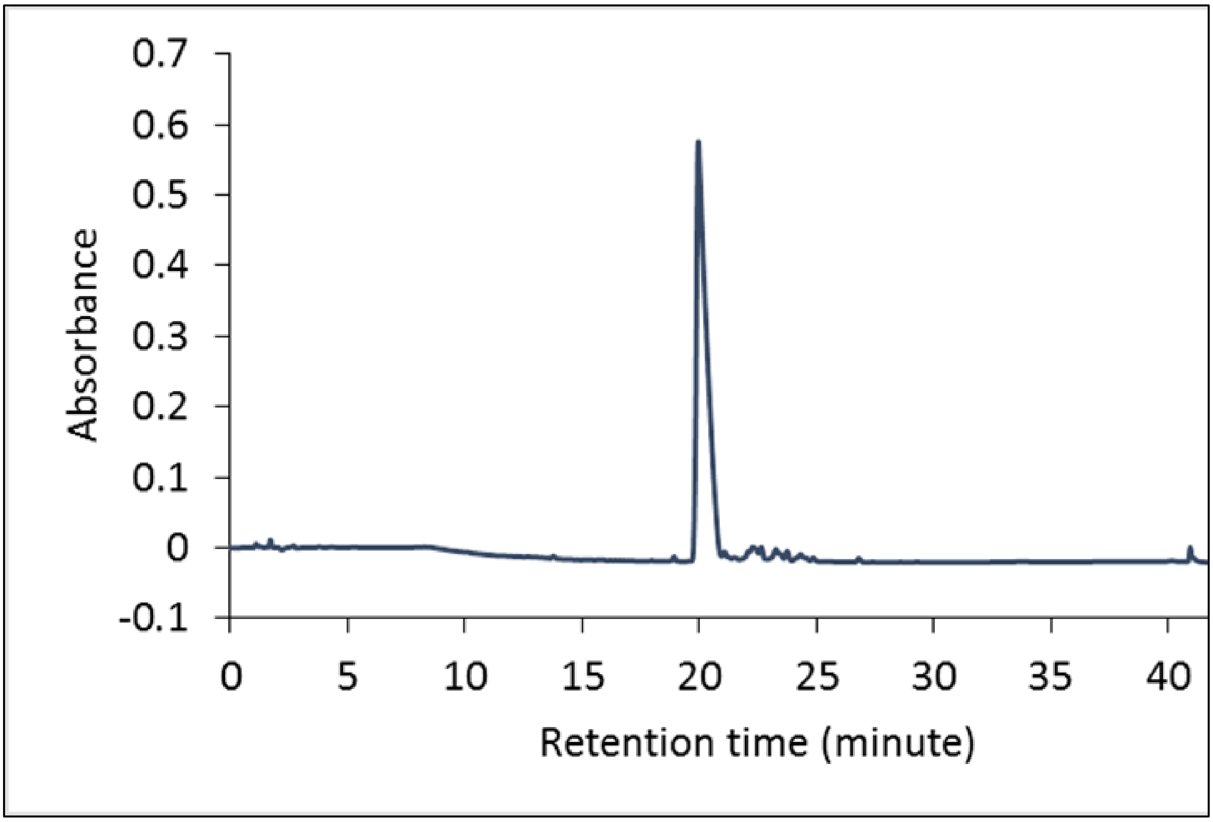

Supplementary Figure S15. Analytical HPLC trace of **PAH3** at 220 nm. Gradient; MeCN (0.1% TFA): H2O (0.1% TFA), 2:98 to 98:2 over 40 min, 1.0 mL min^{-1}.

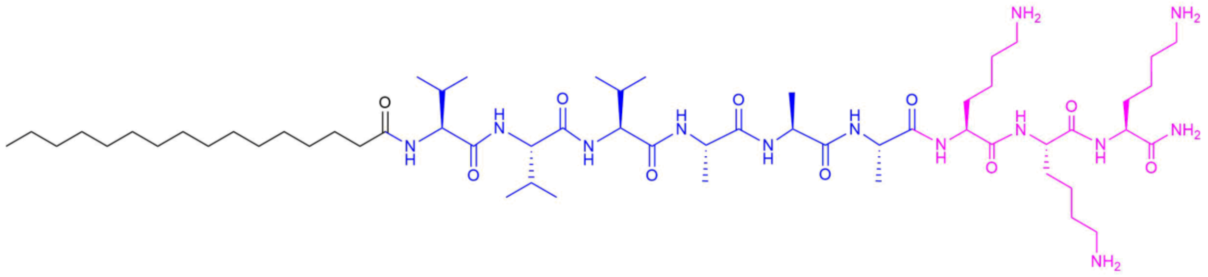

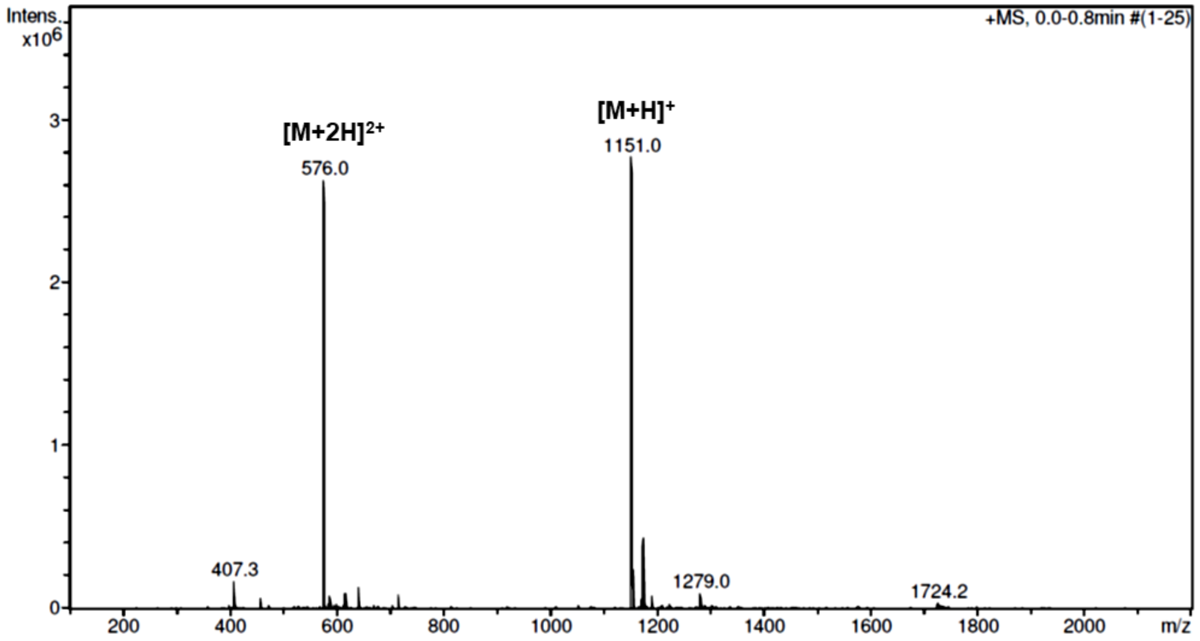

Supplementary Figure S16. ESI-MS of **PAK3** ions $[M+2H]^{2+}$ and $[M+H]^{+}$.

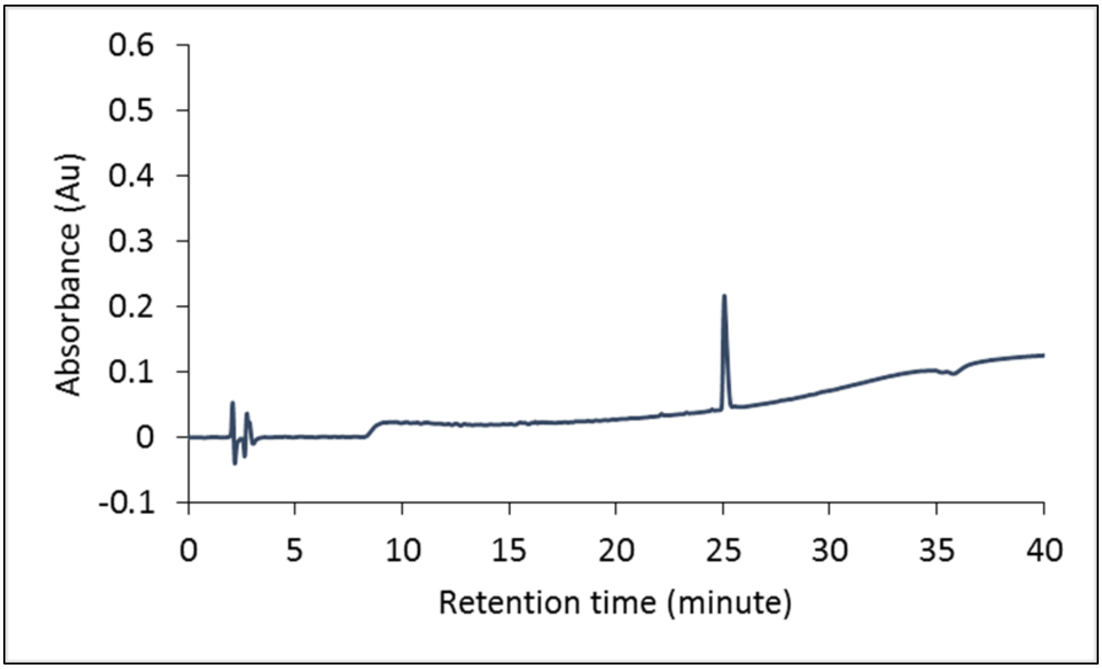

Supplementary Figure S17. Analytical HPLC trace of **PAK3** at 220 nm. Gradient; MeCN (0.1% TFA): H2O (0.1% TFA), 2:98 to 98:2 over 40 min, 1.0 mL min^{-1}.

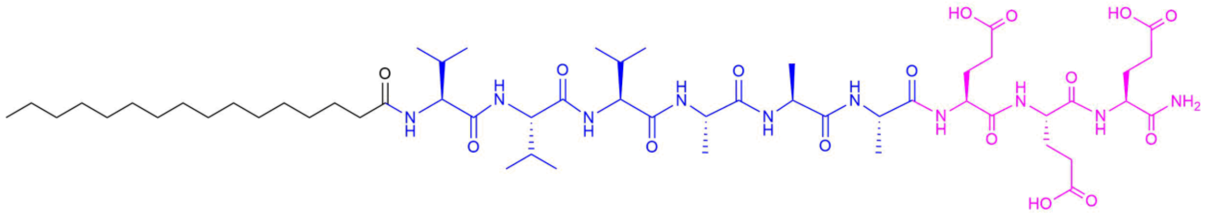

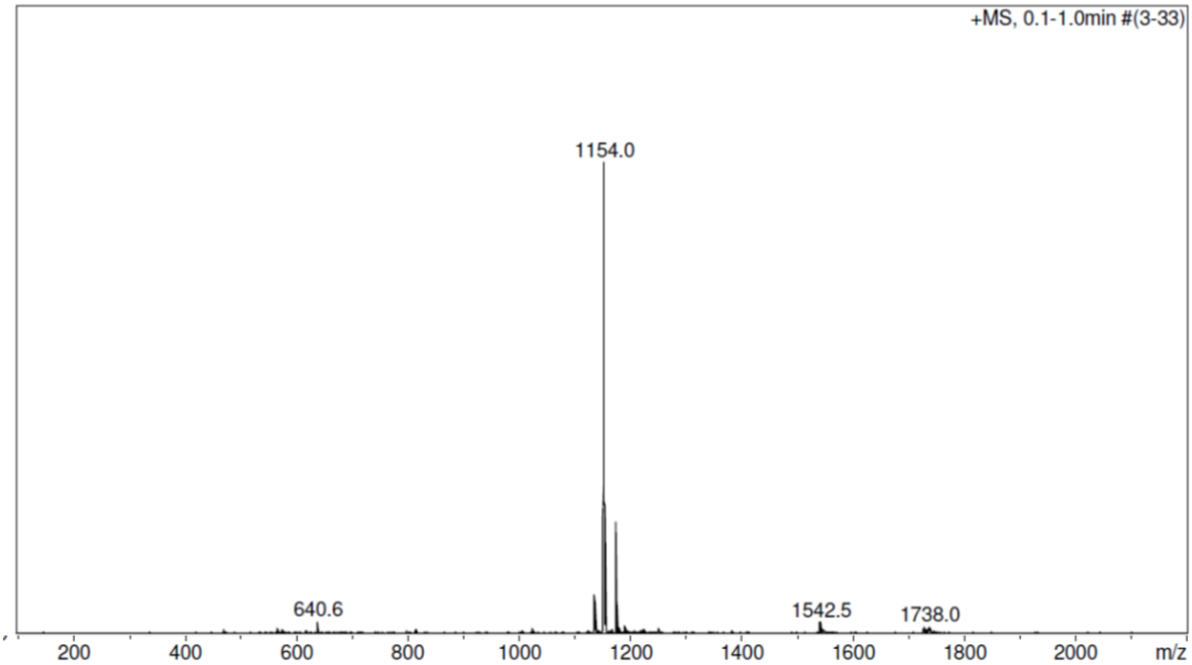

Supplementary Figure S18. ESI-MS of **PAE3** ions $[M+H]^+$.

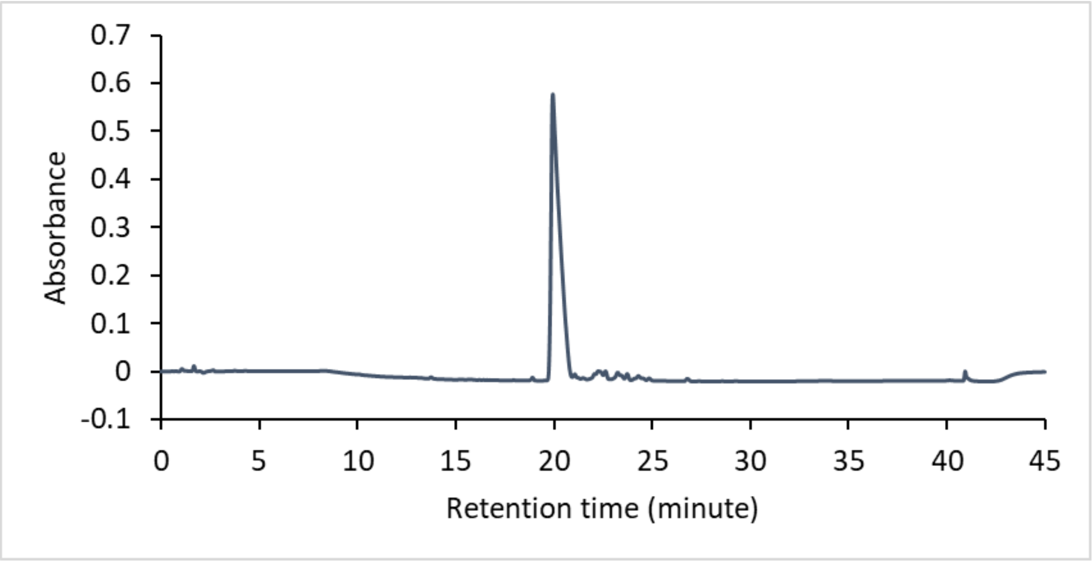

Supplementary Figure S19. Analytical HPLC trace of **PAE3** at 220 nm. Gradient; MeCN : H2O (0.1% NH_4OH), 2:98 to 98:2 over 45 min, 1.0 mL min^{-1}.

References

1. A. Nelson and T. Cosgrove. A Small-Angle Neutron Scattering Study of Adsorbed Polyethylene Oxide on Laponite. *Langmuir,* **2004**, 20, 2298-2304.
2. S. Elsharkawy, M. Al-Jawad, M. F. Pantano, E. Tejeda-Montes, K. Mehta, H. Jamal, S. Agarwal, K. Shuturminska, A. Rice, N. V. Tarakina, R. M. Wilson, A. J. Bushby, M. Alonso, J. C. Rodriguez-Cabello, E. Barbieri, A. Del Río Hernández, M. M. Stevens, N. M. Pugno, P. Anderson and A. Mata. Protein Disorder–Order Interplay to Guide the Growth of Hierarchical Mineralized Structures. *Nat. Commun.* 2018, **9**, 2145-2145.
3. G. Cidonio, C.R. Alcala-Orozco, K.S. Lim, M. Glinka, I. Mutreja, Y.-H. Kim, J.I. Dawson, T.B.F. Woodfield, R.O.C. Oreffo. Osteogenic and Angiogenic Tissue Formation in High Fidelity Nanocomposite Laponite-Gelatin Bioinks. *Biofabrication*. **2019**, 11, 035027.
4. T. Kawamoto and M. Shimizu. A Method for Preparing 2- to 50-µm-Thick Fresh-Frozen Sections of Large Samples and Undecalcified Hard Tissues. *Histochem. Cell Biol*. **2000**, 113, 331–339.
